# Efficacy and safety of oral Chinese medicine combined with chemotherapy: a systematic review and network meta-analysis

**DOI:** 10.3389/fphar.2025.1579613

**Published:** 2025-06-12

**Authors:** Yuqi Ma, Jia Li, Liyun Liu, Tao Shen

**Affiliations:** School of Basic Medical Sciences, Chengdu University of Traditional Chinese Medicine, Chengdu, China

**Keywords:** non-small-cell lung cancer, traditional Chinese medicine, network meta-analysis, oral Chinese medicine, safety

## Abstract

**Background:**

Non-small-cell lung cancer (NSCLC), a leading cause of global cancer mortality, often presents at advanced stages with limited efficacy from standard chemotherapy. This study evaluated the efficacy and safety of six oral traditional Chinese medicines (TCMs) combined with chemotherapy for NSCLC.

**Methods:**

Following PRISMA-NMA guidelines, a systematic review and network meta-analysis of 36 randomized controlled trials (RCTs, *n* = 2,846 patients) was conducted. Databases including PubMed, China National Knowledge Infrastructure (CNKI), and Web of Science were searched. Outcomes assessed included objective response rate (ORR), immune markers (CD4–CD8 ratio and natural killer (NK) cells), tumor markers (CA125, carcinoembryonic antigen (CEA), and CYFRA21-1), and adverse events. Data were synthesized using STATA 14.0 and R software, with risk of bias evaluated via the Cochrane RoB 2.0 tool.

**Results:**

Compared to chemotherapy alone, Tongguanteng (TGT, *Marsdenia tenacissima*) demonstrated superior improvement in the ORR [OR = 1.88, 95% CI: 1.25–2.83]. This effect may be attributable to its vincristine content, which modulates apoptosis through cell-cycle regulation pathways. Huisheng (HS) ranked second in efficacy [OR = 1.34, 95% CI: 1.10–1.61], with its emodin component suppressing NSCLC proliferation via NF-κB pathway inhibition. HS was also associated with improvements in immune markers, including CD4+/CD8+ ratio and NK cell activity. Conversely, TGT significantly reduced tumor markers: CA125, CEA, and cytokeratin-19 fragment (CYFRA21-1). This latter observation may be explained by tenacissoside’s inhibition of cytochrome P450 enzymes (CYP2D6/CYP3A4), which alter drug metabolism. Although TCM–chemotherapy combinations exhibited improved safety profiles compared to chemotherapy alone, the analysis revealed potential publication bias and moderate heterogeneity.

**Conclusion:**

HS and TGT, potentially through their bioactive components, may enhance chemotherapy efficacy in NSCLC by targeting immune and metabolic pathways. However, these conclusions need further verification. Findings should be interpreted cautiously due to potential bias, limited RCT numbers, and the geographical concentration of studies. Future research should isolate compound-specific effects and validate mechanisms in global trials.

## 1 Introduction

Non-small-cell lung cancer (NSCLC) remains a leading cause of global mortality and significantly compromises patient quality of life (Kong et al., 2023; Sung et al., 2021). NSCLC—comprising adenocarcinoma, squamous cell carcinoma, and large cell carcinoma—accounts for approximately 85% of all lung cancer cases. The 5-year survival rate for advanced-stage (III–IV) NSCLC is less than 15% (Qin et al., 2024). Current treatment modalities include radiotherapy, chemotherapy, molecularly targeted therapies, and immune checkpoint inhibitors (Cronin et al., 2022). Due to the lack of effective early screening methods, most patients are diagnosed at advanced stages, contributing to poor prognosis (Ettinger et al., 2021). The 2021 National Comprehensive Cancer Network (NCCN) guidelines recommend high-dose cisplatin or paclitaxel–platinum (TP) combination therapy for advanced NSCLC. However, emerging evidence suggests limited efficacy of these regimens (Herbst et al., 2018; Han et al., 2005; de Jong et al., 2023). Additionally, resistance to immune checkpoint inhibitors (e.g., PD-1/PD-L1 inhibitors) and targeted therapies poses a significant clinical challenge (Guisier et al., 2020; Wang et al., 2022). These limitations underscore the urgent need for integrated therapeutic strategies to improve patient outcomes.

*The Guidelines for the Diagnosis and Treatment of Tumors issued by the Chinese Society of TCM* highlight TCM’s role in oncology, including the use of oral Chinese medicines as adjunctive therapies (Wang et al., 2022). Evidence suggests that oral Chinese medicines can significantly improve survival rates and prognosis in lung cancer patients (Zhao et al., 2019a). Tumor markers (e.g., CEA and CYFRA21-1) and inflammatory biomarkers (e.g., IL-6 and CRP) are recognized as key prognostic indicators for NSCLC (Huai et al., 2023). In clinical practice, oral Chinese medicines are increasingly employed alongside conventional treatments to modulate these biomarkers, demonstrating the potential to enhance therapeutic outcomes (Wang et al., 2024; Zhao, 2023a). In TCM theory, NSCLC is classified as pulmonary retention (肺积), a condition linked to blood stasis and qi stagnation. Oral TCM therapies aim to resolve stasis and restore physiological balance through compounds with bioactive properties (Song et al., 2023). For example, astragaloside IV (derived from *Astragalus membranaceus*) reverses paclitaxel resistance in NSCLC by suppressing the EREG/ErbB/ERK signaling pathway, while *Celastrus orbiculatus* extracts induce ROS-mediated pyroptosis and autophagy to inhibit tumor progression (Xiu et al., 2024; Luo et al., 2023). Synergistic interactions among TCM components further amplify their antitumor efficacy (Qin et al., 2020). Currently, multiple standardized oral Chinese medicines are commercially available. This study evaluates the efficacy and safety of six oral traditional Chinese medicines (TCMs) combined with chemotherapy in NSCLC treatment.

## 2 Methods

This study has been registered in the PROSPERO international prospective register of systematic reviews (registration ID: CRD42024580740). The methodology and reporting adhere rigorously to the Preferred Reporting Items for Systematic Reviews and Meta-Analyses extension for Network Meta-Analyses (PRISMA-NMA) guidelines and meta-analysis standards (Hutton et al., 2015).

### 2.1 Literature retrieval

We searched seven databases, namely, China National Knowledge Infrastructure (CNKI), Wanfang Database, China Science and Technology Journal Database (VIP), China Biomedical Literature Database (CBM), PubMed, Embase, and Web of Science, from inception to 17 September 2024. The search approach is explained in Supplementary Material S1A. In addition, the ingredients of the oral Chinese medicine are listed in Supplementary Material S2.

### 2.2 Inclusion criteria

#### 2.2.1 Types of studies

This study includes RCTs published in English or Chinese that examined oral Chinese medicine in conjunction with chemotherapy drugs, namely, cisplatin or cisplatin plus paclitaxel.

#### 2.2.2 Types of participants

Participants were diagnosed in accordance with the Chinese Guidelines for the Diagnosis and Treatment of Common Malignant Tumors and confirmed to have NSCLC by pathology or cytology (Association, 2022). Participants’ ages varied from 35 to 85 years. The treatment duration ranged from 3 to 24 weeks, irrespective of nationality or gender.

#### 2.2.3 Interventions and comparison

The intervention group received treatment with a TCM oral solution in combination with cisplatin or cisplatin plus paclitaxel, whereas the control group was solely treated with cisplatin or cisplatin plus paclitaxel.

#### 2.2.4 Outcome

The primary indicator is the objective response rate (OOR), while the secondary indicators include the TCM syndrome score, CD4–CD8 ratio, NK cells, CYFRA21-1, platelets, CA125, and CEA.

### 2.3 Exclusion criteria

Exclusion criteria were as follows: 1. duplicated literature; 2. literature that used multiple herbal oral medicines; 3. literature that used chemotherapeutic agents other than cisplatin and paclitaxel; and 4. studies employing non-standardized or modified formulations (e.g., decoctions with variable ingredients).

### 2.4 Study selection

EndNote X9 is used to manage all articles. Following the removal of duplicates, two reviewers (YQM and JL) evaluated the papers according to the qualifying criteria. The complete text was subsequently reviewed for evaluation. Disputes were settled by team deliberation or LYL and TS.

### 2.5 Data extraction

YQM and JL, two reviewers, used a pre-made extraction form to extract data. The author’s name, sample size, stage, age, gender (M/F), length of therapy, and outcome indicators were among the information that was retrieved. Two reviewers cross-checked the extracted data. During the study selection process, disagreements between the two investigators were resolved by LYL and TS.

### 2.6 Assessment of risk of bias

RoB 2.0 (Sterne et al., 2019) was used to assess the risk of bias for the included studies. RoB 2.0 evaluates the risk of bias in five areas, namely, bias from outcome measures, bias from missing outcome data, bias from divergence from established interventions, bias from the randomization method, and bias from selective reporting of outcomes.

### 2.7 Statistical analysis

Statistical analyses were performed using STATA 14.0, R software, and Microsoft Excel 2019. R software (version 4.3.0) was used for data synthesis. MD and 95% CI were measured for continuous variables, and relative risk (RR) was calculated for the 95% CI of dichotomous data. For dichotomous variables, RR was used as an effect size indicator based on the OOR rate of patients with a 95% CI for reporting (Dias et al., 2013; Mills et al., 2013). For continuous variables, such as NK cells, CA125, CEA, CYFRA21-1, and CD4–CD8, MD and 95% CI were calculated. Comparisons were made using the SUCRA for TCM oral liquids. SUCRA curves indicated the most effective and least effective treatments in terms of 100% and 0% percentages, respectively. SUCRA curves and consistency tests were performed using Stata 14.0 software (Rücker and Schwarzer, 2015; Trinquart et al., 2016). Funnel plots were drawn and compared to determine whether there was a publication bias in this network meta-analysis.

Subsequently, time series analysis (TSA) was implemented to evaluate both data robustness and meta-analytic conclusions. This methodology employs sequential monitoring boundaries through the calculation of the accrued sample size (information size, IS) from included studies. The Z-curve represents the temporal accumulation of trial evidence, where intersection with alpha-spending boundaries indicates conclusive evidence for intervention efficacy, while crossing futility boundaries demonstrates therapeutic ineffectiveness. TSA boundaries mitigate type I (α = 5%) and type II (β = 20%) error risks through predefined monitoring criteria. Our analysis preserved a two-sided α = 5% significance level and calculated the required IS based on 80% power to detect a 20% relative risk reduction in mechanical compression. Analytical procedures used Copenhagen Trial Unit’s TSA software (version 0.9.5.10 Beta9).

## 3 Results

### 3.1 Study selection and characteristics

**FIGURE 1:**
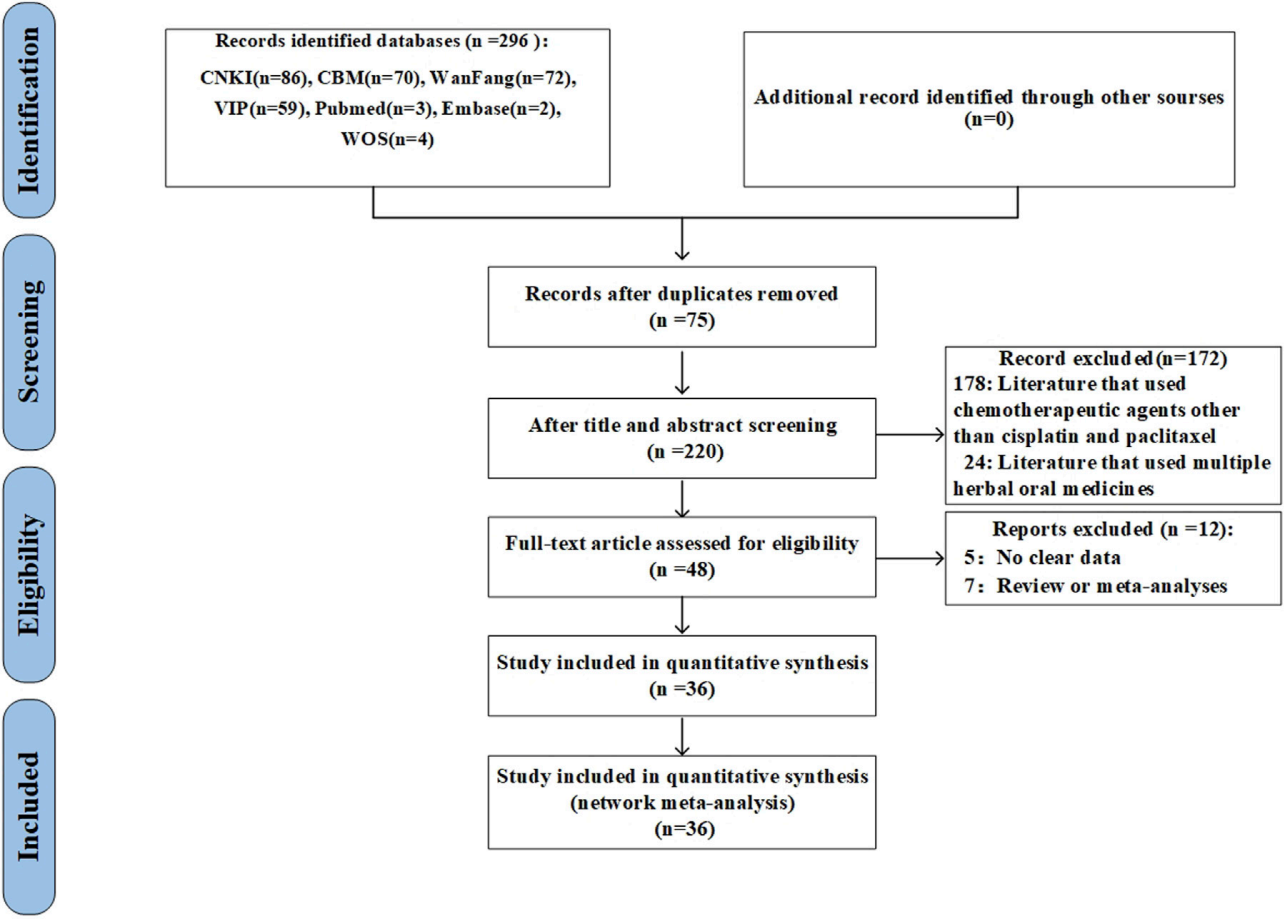
Flow chart.

The preliminary analysis revealed 296 studies through the search approach. Following the elimination of duplicates, 220 studies were evaluated based on their titles and abstracts. At this juncture, 172 extraneous citations were eliminated, allowing for the selection of 48 studies for comprehensive review. Following the exclusion of reviews, meta-analyses, and studies lacking outcome data, 36 studies were incorporated (Figure 1).

### 3.2 Characteristics of included studies

A total of 36 studies involving 2,846 patients were included in this study, covering six oral Chinese medicines, namely, JinFuKang (JFK) oral liquid, Huisheng (HS) oral liquid, Xuekang (XK) oral liquid, Tianfoshen (TFS) oral liquid, Fuzheng (FZ) oral liquid, and Tongguanteng (TGT) oral liquid (Lin, 2003; Chen, 2011; Ding et al., 2011; Liu et al., 2011a; Liu et al., 2011b; Xu et al., 2011; Yu, 2011; Jia, 2013a; Jia, 2013b; Zhang, 2013; Jia, 2014; Wei, 2014; Song, 2015; Li, 2016; Pu, 2016; Yu, 2016; Li, 2017; Liu, 2017; Ranbai, 2017; Wu, 2017; Zhang, 2017; Yang L., 2018; Yang W., 2018; Yang X., 2018; Li, 2019; Mu, 2019; Tang, 2019; Zhai, 2019; Zhang, 2019; Zhao et al., 2019b; Xiao, 2021; Zhang, 2021; Lu, 2022; Liu, 2023; Song, 2023; Xie, 2023; Zhao, 2023b). The retrieval strategies of the included studies can be found in Supplementary Material S1B.

### 3.3 Quality assessment

**FIGURE 2:**
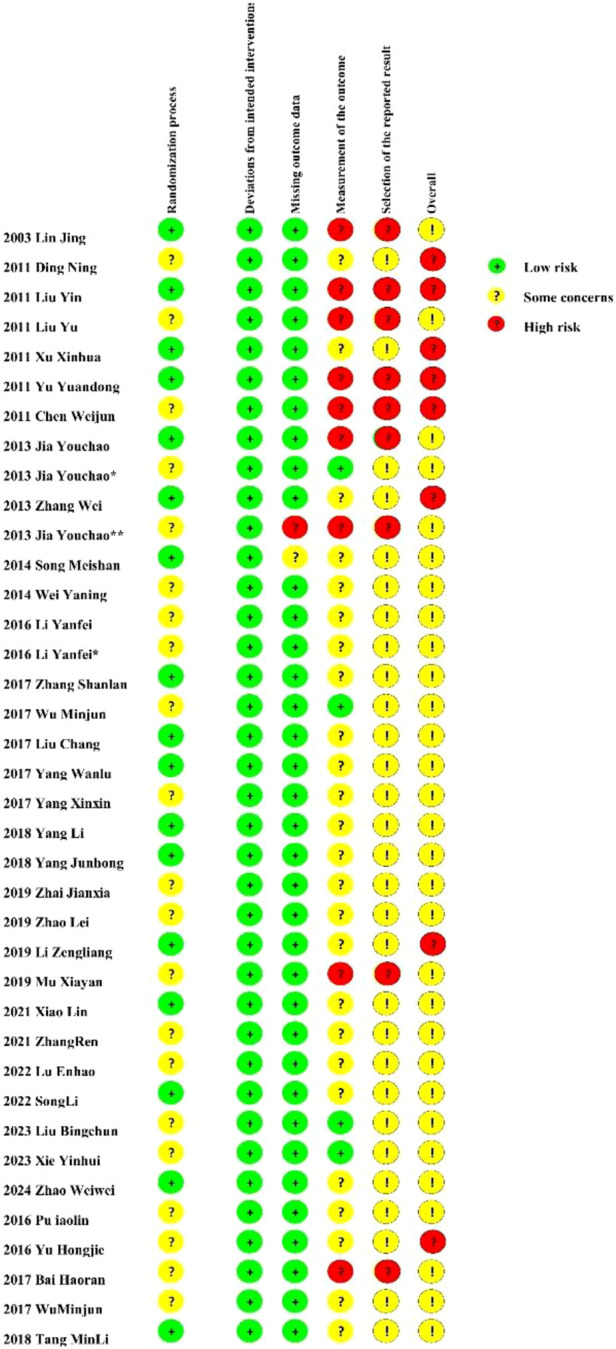
Risk of bias summary.

Out of the 36 randomized controlled trials examined, 16 disclosed their randomization methodologies. Nevertheless, the methodology for randomized sequence creation was ambiguous or inadequately documented in 20 investigations. 9 studies were assessed as high-risk for incompleteness. Detailed quality judgments are presented in Figure 2. In addition, each study includes a ConPhyMP tool sheet (Supplementary Material S4).

### 3.4 Network meta-analysis

**FIGURE 3:**
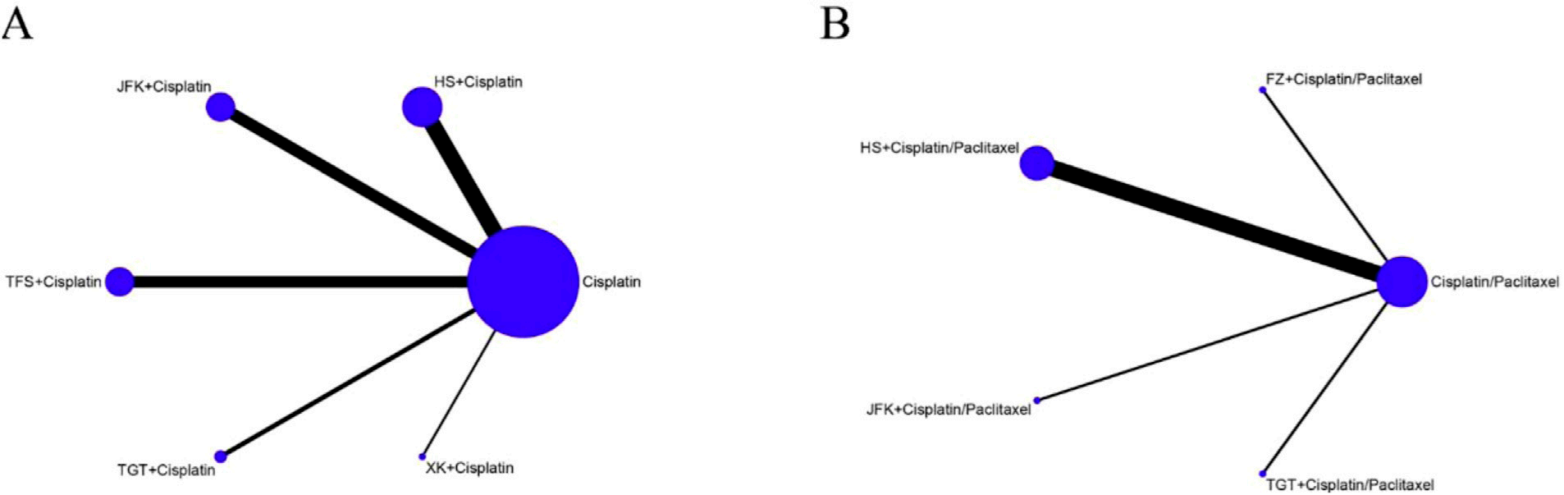
Network graphs of outcomes. **(A)** Objective response rate. **(B)** TP therapy.

The network diagram is shown in Figure 3, where the lines in the graphic represent the interventions of direct comparison, the line thickness indicates the number of studies, and the dot size indicates the sample size of the intervention. We performed a statistical analysis of all indicators using a random-effects model, with a total of 50,000 iterations, beginning with the 20,001st simulation.

### 3.5 Primary indicators

#### 3.5.1 Objective response rate

A total of 30 trials revealed the OOR, encompassing 5 interventions and 2,167 patients. As indicated in Figures 3A, B, the network diagram indicates the relative quantity of available evidence for various therapies, which included JFK, HS, XK, TFS, and TGT oral liquids and chemotherapy. The graphic illustrates 30 direct comparisons of the effects of these therapies on the OOR.

Compared with cisplatin alone, the values were as follows: TGT (OR = 1.88, 95% CI [1.25, 2.83]), HS + cisplatin (OR = 1.34, 95% CI [1.11, 1.61]), JFK + cisplatin (OR = 1.21, 95% CI [0.99, 1.48]), XK + cisplatin (OR = 1.18, 95% CI [0.67, 2.09]), and TFS + cisplatin (OR = 1.07, 95% CI [0.83, 1.37]) (Table 1). The ranking results can be found in Supplementary Material S4A.

**TABLE 1:** Network meta-analysis of the OOR.

XK + cisplatin					
0.63 (0.31, 1.27)	TGT + cisplatin				
1.11 (0.59, 2.07)	1.76 (1.09, 2.85)	TFS + cisplatin			
0.97 (0.53, 1.78)	1.55 (0.98, 2.44)	0.88 (0.64, 1.21)	JFK + cisplatin		
0.88 (0.49, 1.61)	1.40 (0.89, 2.20)	0.80 (0.58, 1.09)	0.91 (0.69, 1.19)	HS + cisplatin	
1.18 (0.67, 2.09)	1.88 (1.25, 2.83)	1.07 (0.83, 1.37)	1.21 (0.99, 1.48)	1.34 (1.11, 1.61)	cisplatin

We also evaluated the effectiveness of adding four oral Chinese medicines to TP therapy. Compared with cisplatin + paclitaxel, the values were as follows: HS + cisplatin/paclitaxel (OR = 1.48, 95% CI [0.70, 3.10]), JFK + cisplatin/paclitaxel (OR = 1.40, 95% CI [1.17, 1.66]), TGT + cisplatin/paclitaxel (OR = 1.27, 95% CI [0.65, 2.48]), and FZ + cisplatin/paclitaxel (OR = 1.17, 95% CI [0.48, 2.86]) (Table 2). The ranking results can be found in Supplementary Material S4B.

**TABLE 2:** Network meta-analysis of TP therapy.

TGT + cisplatin/paclitaxel				
0.86 (0.32, 2.34)	JFK + cisplatin/paclitaxel			
0.91 (0.46, 1.81)	1.06 (0.49, 2.26)	HS + cisplatin/paclitaxel		
1.09 (0.36, 3.34)	1.27 (0.40, 4.05)	1.20 (0.48, 2.99)	FZ + cisplatin/paclitaxel	
1.27 (0.65, 2.48)	1.40 (1.17, 1.66)	1.48 (0.70, 3.10)	1.17 (0.48, 2.86)	Cisplatin/paclitaxel

#### 3.5.2 Subgroup analysis

We evaluated the ORR across varying treatment durations (Supplementary Material S5). In subgroup analyses of cisplatin combined with oral Chinese medicines for NSCLC, inter-subgroup heterogeneity was low (*I* ^2^ < 50%, *p* > 0.05), supporting the application of a fixed-effects model. The results suggest a positive correlation between treatment duration and efficacy, with TGT + cisplatin demonstrating the highest efficacy at 24 weeks (OR = 3.33, 95% CI [1.14, 9.75]).

Similarly, in subgroup analyses of TP therapy combined with TCM oral formulations for NSCLC, heterogeneity remained low (*I* ^2^ < 50%, *p* > 0.05), justifying the use of a fixed-effects model. The analysis identified 3 weeks as the optimal treatment duration, with statistically significant differences (*p* < 0.05) observed, particularly for HS + cisplatin, which showed the highest efficacy (OR = 3.25, 95% CI [1.18, 8.96]). No significant differences in total treatment efficacy were observed among other subgroups (*p* > 0.05).

#### 3.5.3 Time series analysis

The application of time series analysis (TSA) boundaries to favorable ORR outcomes revealed a required information size (IS) of 4,011 to achieve 80% statistical power. Furthermore, ORR analysis for cisplatin–paclitaxel combination therapy indicated that an IS of 6,429 was necessary to attain 80% power. However, the Z-curve crossed the futility boundary even though it failed to cross TSA boundaries (Supplementary Material S6). These pieces of evidence suggested some correlation between interventions and outcomes.

### 3.6 Secondary indicators

#### 3.6.1 TCM syndrome

**FIGURE 4:**
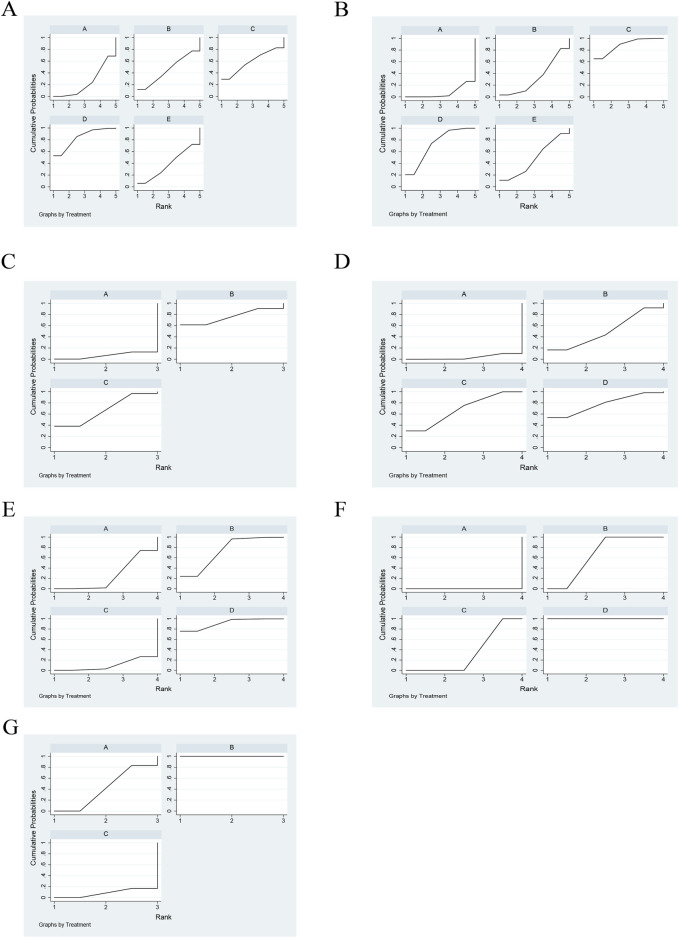
Rank of cumulative probabilities for outcomes. **(A)** TCM syndrome. **(B)** CD4–CD8. **(C)** NK cells. **(D)** CA125. **(E)** CEA. **(F)** CYFRA21-1. **(G)** Platelets.

Ten articles documented TCM syndrome, with a total of 827 cases. The ranking results for TCM evidence frequency ratings were as follows: JFK + cisplatin (83.6%) > HS + cisplatin (59.1%) > FZ + cisplatin (45.2%) > TFS + cisplatin (38.1%) > cisplatin (24%) (Figure 4A; Table 3).

**TABLE 3:** SUCRA results of TCM syndrome.

Intervention	TCM syndrome	
	SUCRA %	Rank
JFK + cisplatin	83.6%	1
HS + cisplatin	59.1%	2
FZ + cisplatin	45.2%	3
TFS + cisplatin	38.1%	4
Cisplatin	24%	5

#### 3.6.2 Immune markers

Immune markers were reported in 14 articles, 10 of which reported CD4–CD8 and four reported NK cells, for a total of 997 patients. The ranking results of CD4–-CD8 were HS + cisplatin (88.5%) > JFK + cisplatin (72.7%) > TFS + cisplatin (48.3%) > FZ + cisplatin (33.5%) > cisplatin (7.1%) (Figure 4B; Table 4). In addition, the ranking results of NK cells were HS + cisplatin (76%) > JFK + cisplatin (67.5%) > cisplatin (6.6%) (Figure 4C; Table 5).

**TABLE 4:** SUCRA of CD4–CD8.

Intervention	CD4–CD8	
	SUCRA %	Rank
HS + cisplatin	88.5%	1
JFK + cisplatin	72.7%	2
TFS + cisplatin	48.3%	3
FZ + cisplatin	33.5%	4
Cisplatin	7.1%	5

**TABLE 5:** SUCRA of NK cells.

Intervention	NK cells	
	SUCRA %	Rank
HS + cisplatin	76%	1
JFK + cisplatin	57.5%	2
Cisplatin	6.6%	3

#### 3.6.3 Tumor markers

Nine articles reported tumor markers, five of which reported CA125, five of which reported CEA, and four of which reported CYFRA21-1, for a total of 804 patients. The ranking results of CA125 were cisplatin + TGT (77.6%) > cisplatin + JFK (68.3%) > cisplatin + HS (50.6%) > cisplatin (3.5%) (Figure 4D; Table 6). The ranking results of CEA were cisplatin + TGT (91.5%) > cisplatin + HS (73.4%) > cisplatin + JFK (25.3%) > cisplatin (9.8%) (Figure 4E; Table 7). Additionally, the results of CYFRA21-1 were ranked as cisplatin + TGT (100%) > cisplatin + HS (66.7%) > cisplatin + JFK (33.3%) > cisplatin (0%) (Figure 4F; Table 8).

**TABLE 6:** SUCRA of CA125.

Intervention	CA125	
	SUCRA %	Rank
TGT + cisplatin	77.6%	1
JFK + cisplatin	68.3%	2
HS + cisplatin	50.6%	3
Cisplatin	3.5%	4

**TABLE 7:** SUCRA of CEA.

Intervention	CEA	
	SUCRA %	Rank
TGT + cisplatin	91.5	1
HS + cisplatin	73.4%	2
JFK + cisplatin	25.3%	3
Cisplatin	9.8%	4

**TABLE 8:** SUCRA of CYFRA21-1.

Intervention	CYFRA21-1	
	SUCRA %	Rank
TGT + cisplatin	100%	1
HS + cisplatin	66.7%	2
JFK + cisplatin	33.3%	3
Cisplatin	0.0%	4

#### 3.6.4 Platelet

Seven articles reported platelets for a total of 415 patients. The ranking results of platelets were HS + cisplatin (100%) > XK + cisplatin (41.7%) > cisplatin (8.3%) (Figure 4G; Table 9).

**TABLE 9:** SUCRA of platelets.

Intervention	Platelet	
	SUCRA %	Rank
HS + cisplatin	99.9%	1
XK + cisplatin	41.7%	2
Cisplatin	8.3%	3

### 3.7 Consistency test

Due to the network diagrams not forming closed loops, we conducted a consistency test on the relevant literature. The results reveal that the *p*-values for all indicators were below 0.05 (*p* < 0.05), indicating significant inconsistencies, with the exceptions of TGT in OOR and TP therapy; HS and JFK in CD4–CD8; JFK in TCM syndrome; JFK, TGT, and HS in tumor markers; and HS and XK in platelets (*p* > 0.05) (Supplementary Material S7).

### 3.8 Safety

Eleven publications reported adverse reactions to oral Chinese medicine + cisplatin, and seven publications reported adverse reactions to oral Chinese medicine + TP therapy, all of which were categorized into four classes. Among these, seven publications reported adverse reactions to HS, five to JFK, two to TFS, two to FZ, and one to TGT (Supplementary Material S8A,B).

#### 3.8.1 Publication bias

**FIGURE 5:**
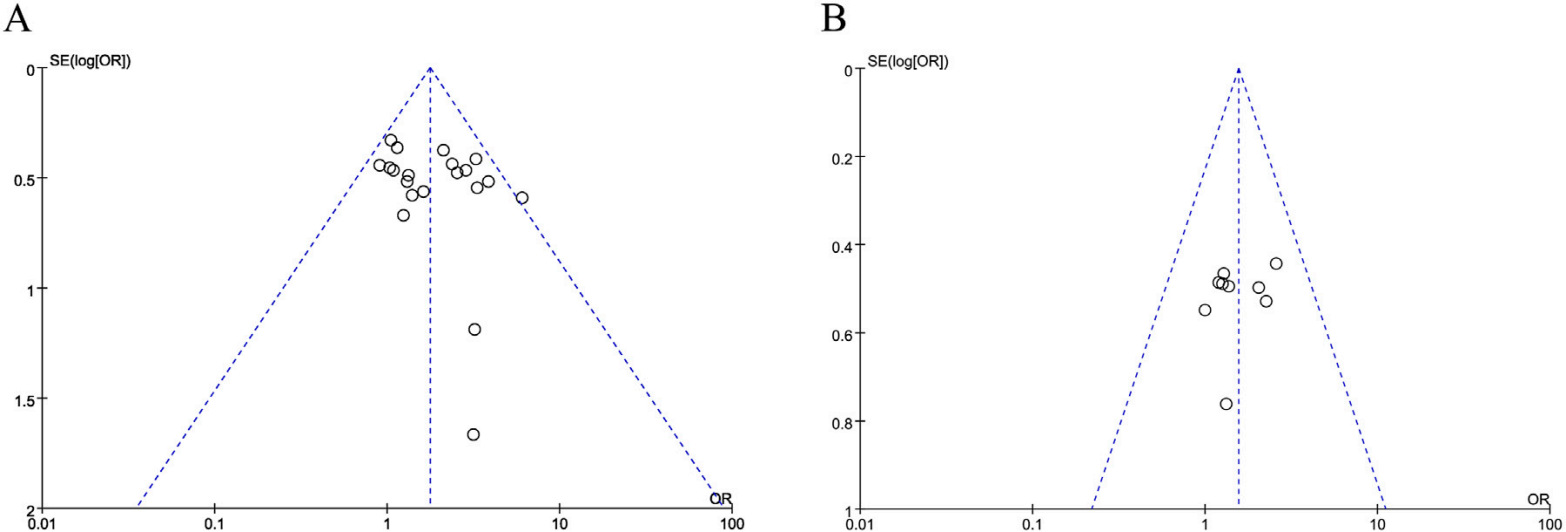
Funnel plots of outcome. **(A)** Objective response rate. **(B)** TP therapy.

The funnel plot of the objective response rates is visibly lopsided, showing publication bias in these data (Figures 5A,B).

#### 3.8.2 Sensitivity analysis

To address potential bias, sensitivity analyses excluded studies with a high risk of bias in randomization or outcome completeness. Subgroup analyses further stratified results by treatment duration, and fixed-effects models were applied to account for heterogeneity.

## 4 Discussion

This network meta-analysis constitutes the first systematic assessment of six oral TCMs combined with chemotherapy for NSCLC. Our findings indicate that adjunctive TCM therapy significantly improves the ORR, modulates immune and tumor biomarkers, reduces TCM syndrome scores, and mitigates chemotherapy-related adverse events. Among the evaluated TCMs, HS and TGT demonstrated the highest therapeutic efficacy. However, the mechanistic foundations, clinical relevance, and limitations of these findings require further investigation.

Regarding the ORR, immune markers (e.g., CD4+/CD8+ ratio and NK cell activity), and platelet regulation, HS and TGT exhibited the most pronounced therapeutic effects. Existing evidence highlights the significant role of HS and TGT components in NSCLC pathogenesis (Wang et al., 2014; Luo et al., 2024; Que et al., 2021). HS primarily contains emodin and eugenol (Xiang and Ay, 2014), both demonstrating mechanistic relevance: emodin suppresses KRAS-mutant NSCLC proliferation by downregulating sPLA2-IIa and NF-κB pathway expression, positioning it as a potential therapeutic target (Zhang et al., 2022), while eugenol exerts antitumor activity via TRIM59-mediated inhibition of NF-κB signaling (Cui et al., 2019). These mechanisms correlate with observed immunomodulatory improvements, suggesting that HS enhances chemotherapy through dual anti-proliferative and immune-enhancing pathways. TGT, enriched with vincristine and tenacissoside, significantly reduced tumor markers (CA125, CEA, and CYFRA21-1) (Wei et al., 2024; M, 2023). Vincristine impedes NSCLC progression by disrupting cell-cycle dynamics and inducing apoptosis (AbouEl et al., 2006; Ahn et al., 2013; Proia et al., 2012), whereas tenacissoside inhibits gefitinib metabolites (M523595, M608236, and M537194) within 24 h post-administration and suppresses hepatic CYP2D6/CYP3A4 activity within 6 h (Zhao et al., 2020). This dual-action mechanism—direct cytotoxicity and metabolic interference—likely explains TGT’s superior tumor marker suppression. To elucidate TCMs’ adjuvant role, future studies should delineate compound-specific mechanisms and validate findings in multinational cohorts.

Safety analyses revealed that TCM–chemotherapy combinations were better tolerated than chemotherapy alone. However, due to limited data, chemotherapy-associated toxicities remain a critical concern requiring vigilant monitoring (Huang et al., 2020).

Despite these insights, our study has notable limitations. First, geographical bias arises from the predominance of Chinese trials, which may limit generalizability to global populations. Second, the observed inconsistencies likely stem from clinical and methodological heterogeneity, particularly sparse direct comparisons and variability in chemotherapy protocols. Although sensitivity analyses support the primary conclusions, these limitations necessitate cautious interpretation. Third, the lack of pharmacokinetic data prevents definitive conclusions about compound-specific interactions. Fourth, the relatively small number of trials for certain interventions, particularly in subgroup analyses (e.g., TP therapy combinations), may limit the robustness of indirect comparisons in the network meta-analysis.

## 5 Conclusion

This study investigated the efficacy and safety of six oral Chinese medicines in conjunction with chemotherapy drugs. HS and TGT may be better oral Chinese medicines for the adjuvant therapy of NSCLC. The results need to be considered with caution due to the potential risk of bias and the small number of RCTs.
