# Correction: Efficacy and safety of oral Chinese medicine combined with chemotherapy: a systematic review and network meta-analysis

**DOI:** 10.3389/fphar.2025.1646824

**Published:** 2025-08-29

**Authors:** Yuqi Ma, Jia Li, Liyun Liu, Tao Shen

**Affiliations:** School of Basic Medical Sciences, Chengdu University of Traditional Chinese Medicine, Chengdu, China

**Keywords:** non-small-cell lung cancer, traditional Chinese medicine, network meta-analysis, oral Chinese medicine, safety

The figure graphics and captions for Figures 2, 3 were reversed in the published article. Figure 2 was incorrectly linked to the Figure 3 graphic, with the caption ‘Network graphs of outcomes. **(A)** Objective response rate. **(B)** TP therapy’, while Figure 3 was incorrectly linked to the Figure 2 graphic, with the caption ‘Risk of Bias Summary’. The in-text citations were correct. The graphic and caption order have now been corrected.

**FIGURE 2:**
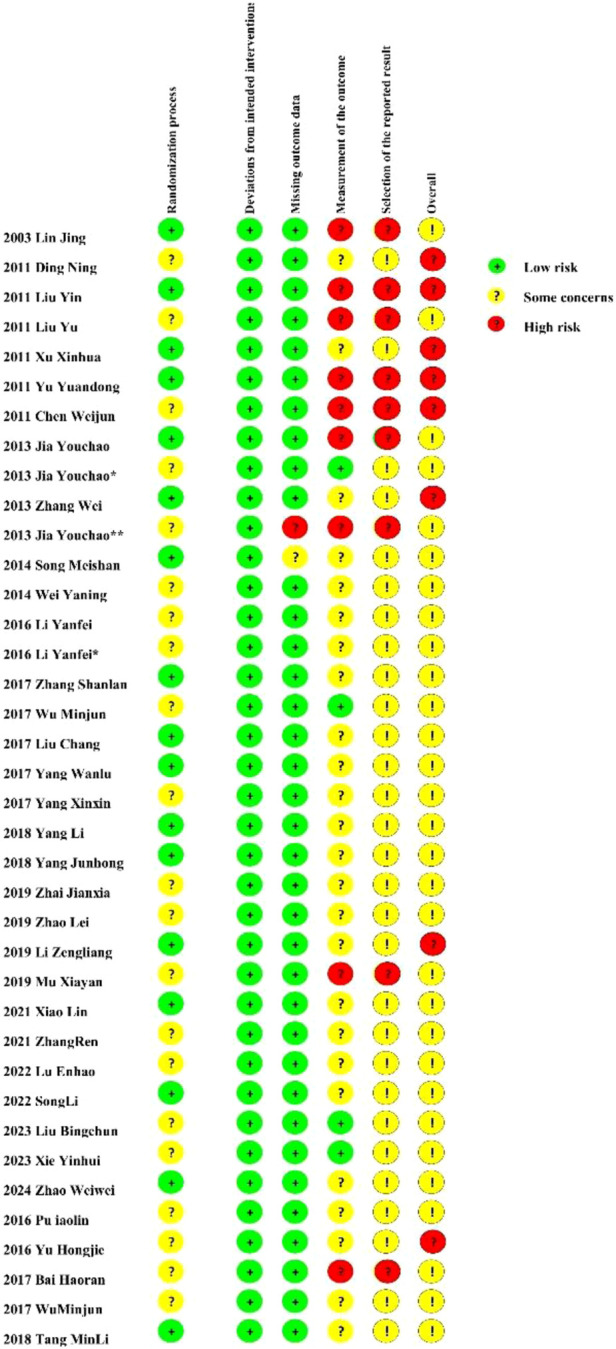
Risk of bias summary.

**FIGURE 3:**
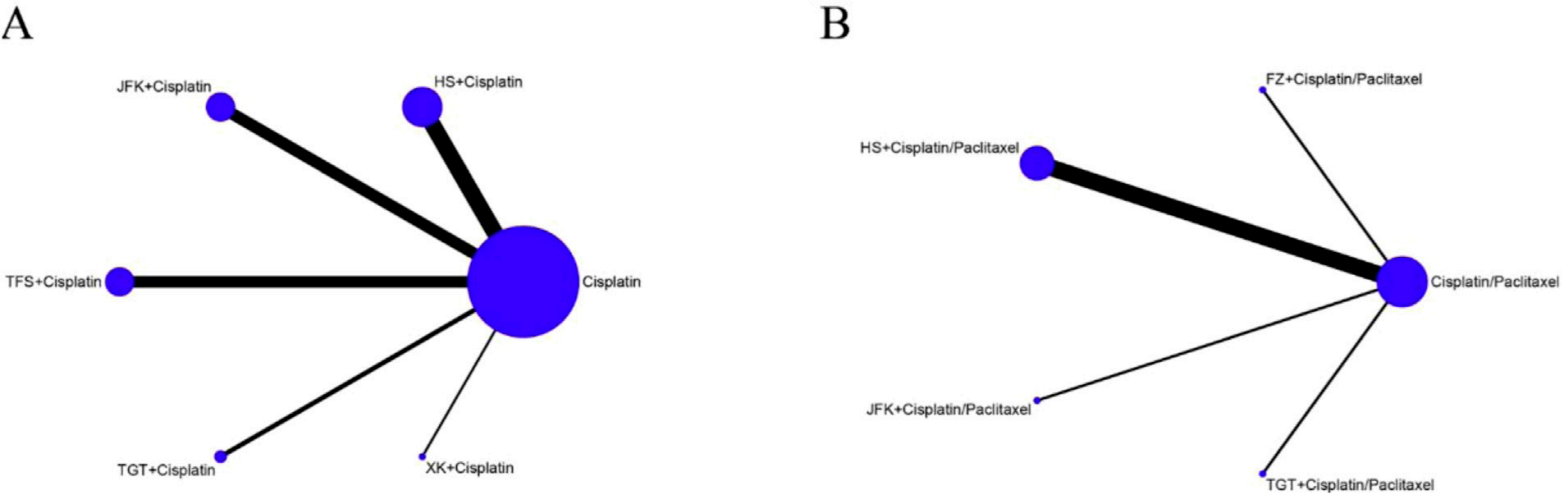
Network graphs of outcomes. **(A)** Objective response rate. **(B)** TP therapy.

The original article has been updated.

