# Supplementary material for: Efficacy and safety of oral Chinese medicine combined with chemotherapy: a systematic review and network meta-analysis
Source: Front Pharmacol. 2025 Jun 12;16:1579613. doi: 10.3389/fphar.2025.1579613 (PMC12198167; doi:10.3389/fphar.2025.1579613)

截止2024年9月17日星期二 22:53pm

知网：86篇
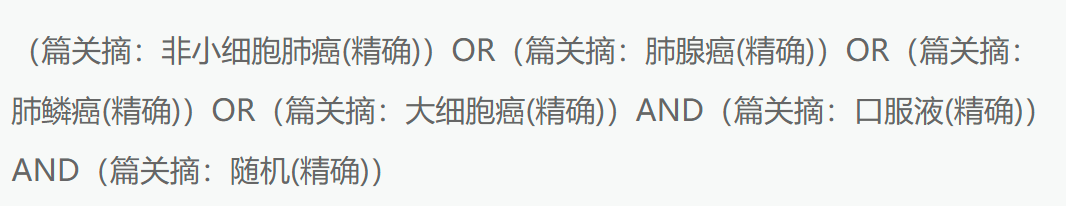


维普：59篇


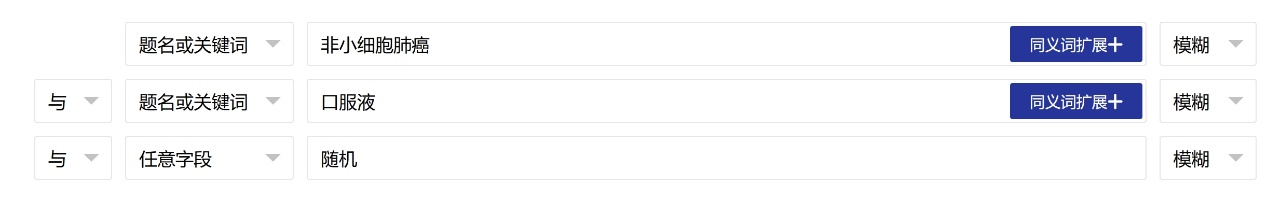


万方：72篇


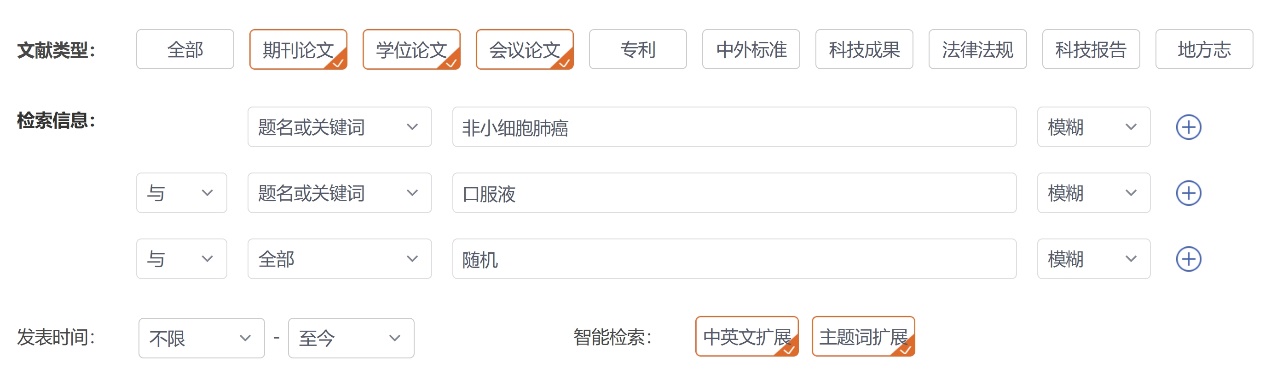


CBM：70篇

("口服液"[常用字段:智能] AND "随机"[全部字段:智能]) AND ("非小细胞肺癌"[常用字段:智能])

Pubmed：3篇

((NSCLC[Title/Abstract]) OR (non small cell lung cancer[Title/Abstract])) AND (TCM Oral Liquid[Title/Abstract])

Embase：2篇


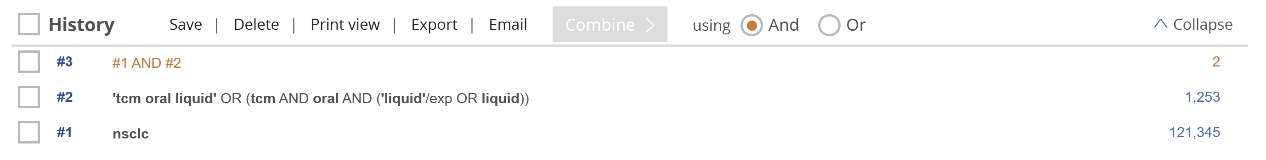


WOS：4篇


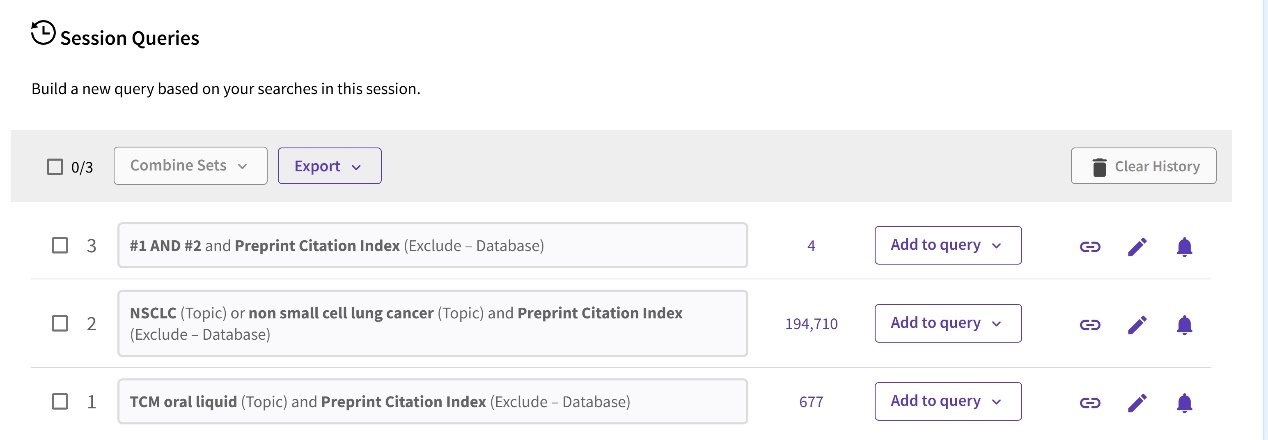

Supplement: Supplementary file 1 [file DataSheet1.zip › Supplementarty Material S3.docx]
