# Supplementary material for: Efficacy and safety of oral Chinese medicine combined with chemotherapy: a systematic review and network meta-analysis
Source: Front Pharmacol. 2025 Jun 12;16:1579613. doi: 10.3389/fphar.2025.1579613 (PMC12198167; doi:10.3389/fphar.2025.1579613)
Supplement: Supplementary file 1 [file DataSheet1.zip › Supplementary Material S1.pdf]

Study	Treatment	Samplesize	Stage	Age	Gender(M/F)	Treatment duration	outcomes
2003 Lin Jing	JFK Oral Liquid + Cisplatin (75mg/m2)<br>Cisplatin (75mg/m2)	27<br>25	IIIA-IV	60(41-69)	20/7<br>17/8	8 weeks	a,c,h
2011 Ding Ning	HS Oral Liquid+Cisplatin (75mg/m2)<br>Cisplatin (75mg/m2)	35<br>32	III-IV	56.5(35-78)	41/26	3 weeks	a,h
2011 Liu Yin	HS Oral Liquid + Cisplatin (80mg/m2) /Paclitaxel(80mg/m2)<br>Cisplatin (80mg/m2) /Paclitaxel(80mg/m2)	35<br>34	III-IV	60(42-70)	46/21	6 weeks	a,h
2011 Liu Yu	XK Oral Liquid + Cisplatin (75mg/m2)<br>Cisplatin (75mg/m2)	25<br>25	III-IV	61.4(48-72)	28/22	3 weeks	g,h
2011 Xu Xinhua	HS Oral Liquid + Cisplatin (80mg/m2) /Paclitaxel(80mg/m2)<br>Cisplatin (80mg/m2) /Paclitaxel(80mg/m2)	35<br>34	IIIA-IV	61.5(42-73)	48/21	3 weeks	a,g
2011 Yu Yuandong	HS Oral Liquid+Cisplatin (75mg/m2)<br>Cisplatin (75mg/m2)	30<br>30	III-IV	56(32-70)	40/20	4 weeks	a,h
2011 ChenWenjun	HS Oral Liquid+Cisplatin (75mg/m2)<br>Cisplatin (75mg/m2)	30<br>26	III-IV	60(39-81)	35/20	4 weeks	h
2013 Jia Youchao	HS Oral Liquid+Cisplatin (75mg/m2)<br>Cisplatin (75mg/m2)	38<br>40	III-IV	62.1(32-67)	23/17<br>24/14	6 weeks	a,c
2013 Jia Youchao*	HS Oral Liquid+Cisplatin (75mg/m2)<br>Cisplatin (75mg/m2)	42<br>40	III-IV	60.4(43-70)	23/17<br>26/14	6 weeks	a,c
2013 Zhang Wei	HS Oral Liquid + Cisplatin (80mg/m2) /Paclitaxel(80mg/m2)<br>Cisplatin (80mg/m2) /Paclitaxel(80mg/m2)	31<br>31	III-IV	52.5(35-70)	37/15	6 weeks	a,h
2014 Jia Youchao	HS Oral Liquid+Cisplatin (75mg/m2)<br>Cisplatin (75mg/m2)	60<br>52	III-IV	63.9<br>66.4	39/23<br>31/19	4 weeks	g
2014 Song Meishan	XK Oral Liquid + Cisplatin (25mg/m2)<br>Cisplatin (75mg/m2)	24<br>24	/	59.8(47-70)<br>61.5(51-73)	21/27	3 weeks	a,g,h
2014 Wei Yaning	HS Oral Liquid + Cisplatin (80mg/m2) /Paclitaxel(80mg/m2)<br>Cisplatin (80mg/m2) /Paclitaxel(80mg/m2)	30<br>30	III-IV	73.5(65-84)	38/22	6 weeks	a,c
2016 Pu Xiaolin	TFS(60mg/d) Oral Liquid + Cisplatin (75mg/m2)<br>Cisplatin (75mg/m2)	30<br>30	IIIB-IV	74(67-81)<br>72(68-78)	17/13<br>15/15	6 weeks	a,c,h
2016 Li Yanfei	XK Oral Liquid + Cisplatin (75mg/m2)<br>Cisplatin (75mg/m2)	22<br>18	/	53.41(43-64)<br>48.44(37-60)	9/13<br>4/14	2 weeks	g,h
2016 Li Yanfei*	XK Oral Liquid + Cisplatin (25mg/m2)<br>Cisplatin (75mg/m2)	20<br>20	/	51.2(33-75)	26/14	2 weeks	g,h
2016 Yu Hongjie	TFS Oral Liquid + Cisplatin (75mg/m2)<br>Cisplatin (75mg/m2)	20<br>20	III-IV	61.93(48-68)<br>64.76(47-69)	12/8<br>13/7	8 weeks	a,h
2017 Zhang Shanlan	TFS Oral Liquid + Cisplatin (75mg/m2)<br>Cisplatin (75mg/m2)	18<br>18	III-IV	52.62(45-76)<br>52.68(47-72)	11/7<br>10/8	6 weeks	a,b,h
2017 Bai Haoran	JFK Oral Liquid+Cisplatin (75mg/m2)<br>Cisplatin (75mg/m2)	41<br>35	IIIB-IV	60.56(40-74)	27/14<br>24/11	8 weeks<br>8 weeks	a,b,c,h
2017 Wu Minjun	HS Oral Liquid+Cisplatin (75mg/m2)<br>Cisplatin (75mg/m2)	48<br>47	III-IV	54.21(36-70)<br>52.17(42-68)	30/18<br>27/20	12 weeks	a,c,h
2017 Liu Chang	TFS Oral Liquid + Cisplatin (75mg/m2) /Paclitaxel(1mg/m2)<br>Cisplatin (75mg/m2) /Paclitaxel(1mg/m2)	76<br>76	III-IV	74(60-81)<br>75(60-80)	41/35<br>40/36	4 weeks	a,h
2017 Yang Wanlu	FZ Oral Liquid + Cisplatin (80mg/m2) /Paclitaxel(80mg/m2)<br>Cisplatin (80mg/m2) /Paclitaxel(80mg/m2)	20<br>20	IIIB-IV	56(49-63)<br>53.75(42-64)	11/9<br>8/12	6 weeks	a,b,c,h
2017 Yuan Xinxin	HS Oral Liquid+Cisplatin (75mg/m2)<br>Cisplatin (75mg/m2)	34<br>34	IIIA-IIIB	62(53-71)<br>63.5(55-70)	15/19<br>18/16	3 weeks	a,h
2018 Tang MinLi	FZ Oral Liquid + Cisplatin (75mg/m2)<br>Cisplatin (75mg/m2)	30<br>30	/	65.03(57-73)<br>62.43(54-71)	16/14<br>18/12	12 weeks	b,h
2018 Yang Li	HS Oral Liquid+Cisplatin (75mg/m2)<br>Cisplatin (75mg/m2)	34<br>34	III-IV	61(42-76)<br>62(42-73)	19/15<br>18/16	6 weeks	a,c,h
2019 Zhang Junhong	JFK Oral Liquid + Cisplatin (75mg/m2)<br>Cisplatin (75mg/m2)	60<br>60	III-IV	54.82(46-72)<br>54.47(45-71)	33/27<br>34/26	8 weeks	a,b,h
2019 Zhai jianxia	HS Oral Liquid + Cisplatin (75mg/m2)<br>Cisplatin (75mg/m2)	52<br>51	III-IV	61.38(47-66)	69/34	9 weeks	a,c,h,i
2019 Li Zengliang	JFK Oral Liquid + Cisplatin (80mg/m2)<br>Cisplatin (80mg/m2)	60<br>60	IIIA-IV	49.81(38-70)<br>49.91(38-71)	31/29<br>32/28	6 weeks	a,c,h
2019 Mu Xiaoyan	JFK Oral Liquid + Cisplatin (75mg/m2) /Paclitaxel(175mg/m2)<br>Cisplatin (75mg/m2) /Paclitaxel(175mg/m2)	44<br>45	IIIA-IV	60.49(51-70)<br>61.17(54-69)	32/12<br>29/16	8 weeks	a,b,c
2021 Xiao Lin	JFK Oral Liquid + Cisplatin(80mg/m2)<br>Cisplatin(80mg/m2)	30<br>30	III-IV	57.56(41-75)<br>57.82(42-78)	20/10<br>18/12	12 weeks	a,c,h,i
2021 Zhang Ren	JFK Oral Liquid + Cisplatin (75mg/m2)<br>Cisplatin (75mg/m2)	61<br>62	III-IV	61(44-72)<br>62(38-75)	53/67<br>57/44	12 weeks	c,i
2022 Lu Enhao	JFK Oral Liquid + Cisplatin (75mg/m2)<br>Cisplatin (75mg/m2)	100<br>98	IB-IIB	61(36-72)<br>62(22-75)	41/59<br>39/59	12 weeks	b
2023 Song Li	TGT Oral Liquid + Cisplatin (75mg/m2) /Paclitaxel(175mg/m2)<br>Cisplatin (75mg/m2) /Paclitaxel(175mg/m2)	43<br>43	III-IV	62.2（37-79）<br>60.1（41-77）	25/18<br>27/17	12 weeks	a,h
2023 Liu Bingchun	TGT Oral Liquid + Cisplatin(80mg/m2)<br>Cisplatin(80mg/m2)	40<br>40	III-IV	58.3(52-65)<br>57.63(53-62)	23/17<br>22/18	9 weeks	a
2023 Xie Yinhui	TGT Oral Liquid + Cisplatin(80mg/m2)<br>Cisplatin(80mg/m2)	30<br>29	III-IV	59.2(45-81)<br>57.8(42-85)	17/13<br>15/14	24 weeks	a,h
2023 Zhao Weiwei	HS Oral Liquid + Cisplatin (75mg/m2) /Paclitaxel(175mg/m2)<br>Cisplatin (75mg/m2) /Paclitaxel(175mg/m2)	30<br>30	IIIB-IV	60.25(54-66)<br>60.89(53-67)	16/14<br>16/12	12 weeks	a,b,c,h,i

a: ORR; b:TCM syndrome score; c: Immune markers; g: Platelet; h: Adverse reactions; i:Tumor markers.
