# Supplementary material for: Efficacy and safety of oral Chinese medicine combined with chemotherapy: a systematic review and network meta-analysis
Source: Front Pharmacol. 2025 Jun 12;16:1579613. doi: 10.3389/fphar.2025.1579613 (PMC12198167; doi:10.3389/fphar.2025.1579613)
Supplement: Supplementary file 1 [file DataSheet1.zip › Supplementary Material S2.docx]

**Supplementary Material**

**Detailed information on** **Oral Chinese medicine**

| **Oral Chinese medicine** | **Source** | **Raw material** | **Phytochemical compositions** | **Therapeutic claims in TCM** | **Indications and Adverse reactions** | **Quality control reported? (Y/N)** | **Chemical analysis reported? (Y/N)** |
| --- | --- | --- | --- | --- | --- | --- | --- |
| JinFukang Oral Liquid (JFK) | Jilin Sanjiu Jinfukang Pharmaceutical Co., Ltd | 1.*ASTRAGALI RADIX PRAEPARATA CUM MELLE*  (Astragalus mongholicus)  2.*DWARF LILYTURF TUBER*  (Ophiopogon japonicus)  3. *ADENOPHORAE RADIX*  (Adenophora triphylla)  4.*COMMON FENUGREEK SEED*  (Trigonella foenum-graecum)  5.*ASIATIC CORNELIAN CHERRY FRUIT*  (Cornus officinalis)  6.*CERA CHINENSIS*  (Ligustrum lucidum)  7. *QIYEYIZHIHUA*  (Paris polyphylla)  8. *EPIMEDII FOLIUM*  (Epimedium sagittatum)  9.*CODONOPSIS RADIX*  (Codonopsis pilosula)  10. *AMUR CORK-TREEE*  (Phellodendron amurense) | Bilobetin,  α-humulene,  pulegone,  isoamyl alcohol and et al. | Tonify Qi and Nourish Yin, Clear Heat and Resolve Toxins​ | ​​Indicated for primary non-small cell lung cancer (NSCLC) with Qi-Yin deficiency syndrome in patients unsuitable for surgery, radiotherapy or chemotherapy, or for combined use with chemotherapy.​  ​​Adverse reactions may include nausea, vomiting, abdominal distension, diarrhea, rash, dizziness, and palpitations.​ | Y - National Food and Drug Administration National Drug Standards | N |
| Huisheng Oral Liquid (HS) | Chengdu Di'ao Group Tianfu Pharmaceutical Co., Ltd | *1.DA HUANG*  (Rheum officinale)  *2.MOTHERWORT HERB* (Leonurus japonicus)  3.*CARTHAMI FLOS*  (Carthamus tinctorius)  4.*HUA JIAO*  (Zanthoxylum bungeanum)  5. *ANGELICAE SINENSIS RADIX*  (Angelica sinensis)  6.*ALLII SATIVI BULBUS*  (Allium sativum)  7. *COMMON BURREED TUBER*  (Sparganium stoloniferum)  8. *ANEMONES RADDEANAE RHIZOMA*  (Anemone raddeana)  9. *CHUANXIONG*  (Conioselinum anthriscoides 'Chuanxiong')  10. *DALBERGIAE ODORIFERAE LIGNUM*  (Dalbergia odorifera)  11. *GINSENG*  (Panax ginseng)  12. *GALANGA GALANGAL FRUIT*  (Alpinia galanga)  13. *CURCUMAE LONGAE RHIZOMA*  (Curcuma longa)  14. *MYRRHA*  (Commiphora myrrha)  15. *KUXINGREN*  (Prunus armeniaca)  16. *PERILLA FRUIT*  (Perilla frutescens)  17. *FENNEL*  (Foeniculum vulgare)  18. *HETAOREN*  (Juglans regia)  19. *CARYOPHYLLI FLOS*  (Syzygium aromaticum)  20. *CORYDALIS RHIZOMA*  (Corydalis yanhusuo)  21. *BAISHAO*  (Paeonia lactiflora)  22. *CATTAIL POLLEN*  (Typha angustifolia)  23. *RUXIANG*  (Boswellia sacra)  24. *EUODIAE FRUCTUS*  (Tetradium ruticarpum)  25. *PINE POLLEN*  (Pinus massoniana)  26. *CASSIA BARK*  (Neolitsea cassia)  27. *BAIZIREN*  (Platycladus orientalis)  28. *DIHUANG*  (Rehmannia glutinosa) | Emodin ， emodin，Eugenol, Evodia rutaecarpa, Salicylic acid sophocarpine, Ginsenoside Re and et al. | Dissolve Masses and Resolve Stasis, Alleviate Pain, Strengthen Immunity​ | ​**​**Indicated for primary hepatocellular carcinoma and lung cancer.​  ​​Possible adverse reactions may include gastrointestinal discomfort, allergic reactions, abnormal liver and kidney function, dizziness and fatigue, and palpitations.​ | Y - National Food and Drug Administration National Drug Standards | N |
| Xuekang Oral Liquid (XK) | Dalian Jingang Pharmaceutical Co., Ltd | 1. *HUANGQI* (Astragalus mongholicus)  2. *GINSENG RADIX ET RHIZOMA*  (Panax ginseng)  3. *WUWEIZI*  (Schisandra chinensis)  4. *ANGELICAE SINENSIS RADIX*  (Angelica sinensis)  5. *BAISHAO*  (Paeonia lactiflora)  6.*GLABROUS GREENBRIER RHIZOME*  (Smilax glabra)  7. *JIXUETENG*  (Spatholobus suberectus)  8. *HESHOUWU*  (Reynoutria multiflora)  9.*ASIATIC CORNELIAN CHERRY FRUIT*  (Cornus officinalis)  10. *DIGUPI*  (Lycium chinense)  11. *CERA CHINENSIS*  (Ligustrum lucidum)  12. *BAIHUASHESHECAO*  (Scleromitrion diffusum)  13.*QIAN CAO*  (Plantago major)  14. *GIANT KNOTWEED RHIZOME*  (Reynoutria japonica)  15.*CHEN PI*  (Citrus reticulata)  16.*BAN XIA*  (Pinellia ternata) | Ferulic acid, Astragaloside IV and et al. | Activate Blood and Resolve Stasis, Reduce Swelling and Dissipate Nodules, Cool Blood and Stanch Bleeding​ | ​​Indicated for leukopenia, thrombocytopenia, and as adjuvant therapy following radiotherapy or chemotherapy.​  ​​Possible adverse reactions may include gastrointestinal discomfort, allergic reactions, dizziness, and other side effects.​ | Y - National Food and Drug Administration National Drug Standards | N |
| Tianfoshen Oral Liquid (TFS) | Changshu Leiyunshang Pharmaceutical Co., Ltd | 1.*CASSIAE SEMEN*  (Senna tora)  2. *CYPERI RHIZOMA*  (Cyperus rotundus)  3.*GAN JIANG*  (Zingiber officinale)  4. *CASSIA BARK*  (Neolitsea cassia)  5.*BAI ZHU*  (Atractylodes macrocephala )  6. *BAISHAO*  (Paeonia lactiflora)  7. *HUANGQI* (Astragalus mongholicus)  8.*DAN SHEN*  (Salvia miltiorrhiza)  9.*DALBERGIAE ODORIFERAE LIGNUM*  (Dalbergia odorifera)  10.*CITRI SARCODACTYLIS FRUCTUS*  (Citrus × limon) | PQ-2,  Cinobufotalin,  Quercetin,  and et al. | Nourish Yin and Tonify Qi, Resolve Toxins and Dissipate Masses​ | Indicated for patients with NSCLC.​  May cause elevated blood pressure, palpitations, insomnia, skin rash, and gastrointestinal discomfort, among other adverse effects.​ | Y - National Food and Drug Administration National Drug Standards | N |
| Fuzheng Oral Liquid (FZ) | Lizhu Group Limin Pharmaceutical Factory | 1*.DANSHEN*  (Salvia miltiorrhiza)  2. *CARTHAMI FLOS*  (Carthamus tinctorius)  3.*CHUAN XIONG*  (Conioselinum anthriscoides 'Chuanxiong')  4. *BAISHAO*  (Paeonia lactiflora)  5. *CORYDALIS RHIZOMA*  (Corydalis yanhusuo)  6. *CHINESE WOLFERRY ROOT-BARK*  (Lycium chinense)  7.*CAULIS POLYGONI MULTIFLORI*  (Reynoutria multiflora)  8. *CYPERI RHIZOMA*  (Cyperus rotundus) | Astragaloside IV, Isoflavone-7-O - β - D-glucoside, Codonopsis pilosula alkynyl glycoside, Lufenamide  and et al. | ​​Promote Qi and Activate Blood, Clear Heat and Resolve Stagnation, Eliminate Accumulations | **​​**Indicated for adjunctive therapy in cases of general malaise, post-operative recovery, or chronic diseases.  **​​**Possible adverse reactions may include fever, excessive internal heat, and gastrointestinal discomfort.​ | Y - National Food and Drug Administration National Drug Standards | N |
| Tongguanteng Oral Liquid (TGT) | Yangling Kesen Biopharmaceutical Co., Ltd | *MARSDENIAE TENACISSIMAE CAULIS*  (Gongronemopsis tenacissima) | Tenacissoside H,  Tenacissoside G,  Tenacissoside I,  Vincristine  and et al. | Clear Heat and Detoxify, Reduce Swelling and Dissipate Nodules, Drain Dampness and Relieve Jaundice, Activate Blood and Resolve Stasis, Enhance Immune Function​ | Indicated for various malignant diseases including esophageal cancer, gastric cancer, hepatocellular carcinoma, colorectal cancer, cervical cancer, and leukemia.​  ​**​**Possible adverse reactions may include fever, drug eruption, gastrointestinal discomfort, arthralgia, and abnormal liver function, among others.​ | Y - National Food and Drug Administration National Drug Standards | N |

**References**

Chen SS, Chen JL, Xie RF, et al. Network pharmacology-based mechanism of Jinfukang Oral Liquid in treating non-small cell lung cancer. Chinese Traditional Patent Medicine. 2019;41(7):1547-55.

Wu GJ, Huang MF, Qi A, et al. Clinical efficacy of Jinfukang Oral Liquid combined with thymosin α1 in non-small cell lung cancer patients with qi-yin deficiency syndrome. Chinese Traditional Patent Medicine. 2025;47(3):790-795.

Zhang R, Zhu LH, Chen ZW, et al. Clinical observation of Jinfukang Oral Liquid combined with adjuvant chemotherapy in postoperative patients with early to mid-stage non-small cell lung cancer with qi-yin deficiency syndrome. Shanghai Journal of Traditional Chinese Medicine 2025;59(2):55-59.

Xiang WJ, Chen AY. Identification of main components in Huisheng Oral Liquid. Shandong Chemical Industry. 2014;43(12):70-4.

Zhao WW. Efficacy and safety of Huisheng Oral Liquid combined with chemotherapy and immunotherapy in modulating PD-1/PD-L1 signaling pathway for advanced non-small cell lung cancer. Hebei Medical Journal. 2023;45(23):3535-3539, 3544.

Zhang HT, Ge Y. Determination of Astragaloside IV in Shengxuekang Oral Liquid. Scientific and Technological Innovation. 2017;(18):30.

ZHAO Y, WU D. Determination of Ferulic Acid in Shengxuekang Oral Liquid by HPLC. Scientific and Technological Innovation. 2016;(08):77.

Cai DF. Therapeutic effect of Naoxuekang Oral Liquid on hypertensive cerebral hemorrhage. Guide of China Medicine. 2015;13(9):219-220.

He JS. A Class III Chinese medicine for reducing toxicity in cancer therapy: Xuekang Oral Liquid. Xiamen Oriental Medicine Research Center, Fujian Province; 2002 Jan 1.

Sun LH. Pharmacodynamic study on anti-colon cancer effects and mechanism exploration of Tianfushen Oral Liquid. Nanjing University of Chinese Medicine.

WANG HL, ZHANG XM, JIAO ZH, et al. Optimization of Extraction Process for Shegan Fuzheng Oral Liquid by Orthogonal Test. Western Journal of Traditional Chinese Medicine. 2012;25(02):23-24.

YANG N, SUN TH, HUANG FH, et al. Simultaneous determination of main components in Shenqi Fuzheng Injection and its pharmacokinetics in rat plasma by UPLC-MS/MS. Chinese Journal of Hospital Pharmacy. 2018;38(12):1250-1255.

Yu M, Tian YX, Tang CH, et al. Clinical study of Tianfoshen Oral Liquid in treating non-small cell lung cancer with qi-yin deficiency syndrome. Chinese Journal of Clinical Oncology and Rehabilitation. 2018;25(3):257-261.

Zong SZ, Wang XY, Wang HB, et al. Determination of five metal elements in Tianfoshen Oral Liquid and its raw materials by flame atomic absorption spectrometry. Physical Testing and Chemical Analysis(Part B:Chemical Analysis). 2011;47(12):1433-1435.

SONG L, ZHANG CX, XIAO Y, et al. Clinical efficacy of Tongguanteng Oral Liquid combined with chemotherapy in treatment of non-small cell lung cancer. Liaoning Journal of Traditional Chinese Medicine. 2023;50(06):138-140.

Zeng BR, Wang LH, Sun YH. Effect of Fuzheng Oral Liquid on chemotherapy-induced toxicity in stage III-IV lung cancer patients with qi-yin deficiency syndrome. Chinese Journal of Information on Traditional Chinese Medicine. 2013;20(12):73-74.

WANG M. Molecular mechanism of Tongguanteng Oral Liquid and its active ingredient kaempferol inhibiting gastric cancer cell proliferation via PI3K/AKT pathway. Gansu University of Chinese Medicine.2023.

Song L, Zhang CX, Xiao Y, et al. Clinical observation of Tongguanteng Oral Liquid combined with chemotherapy for mid-late stage esophageal cancer. Liaoning Journal of Traditional Chinese Medicine.2023;50(3):89-91.
