# Supplementary material for: Efficacy and safety of oral Chinese medicine combined with chemotherapy: a systematic review and network meta-analysis
Source: Front Pharmacol. 2025 Jun 12;16:1579613. doi: 10.3389/fphar.2025.1579613 (PMC12198167; doi:10.3389/fphar.2025.1579613)
Supplement: Supplementary file 1 [file DataSheet1.zip › Supplementary Material S5.docx]

1. **OOR:**

**
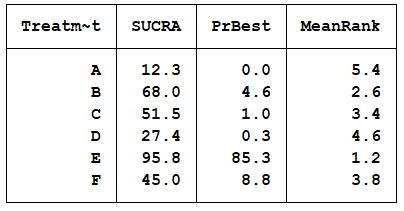
**

A = Cisplatin, B = HS+Cisplatin, C = JFK+Cisplatin,

D = TFS+Cisplatin，E = TGT+Cisplatin, F = XK+Cisplatin

**b. TP therapy:**

**
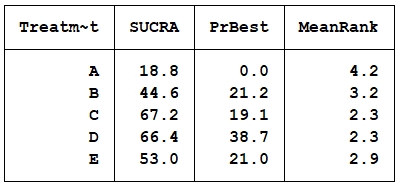
**

A = Cisplatin/Paclitaxel, B = FZ+Cisplatin/Paclitaxel, C = HS+Cisplatin/Paclitaxel, D = JFK+Cisplatin/Paclitaxel, E = TGT+Cisplatin/Paclitaxel
