# Supplementary material for: Efficacy and safety of oral Chinese medicine combined with chemotherapy: a systematic review and network meta-analysis
Source: Front Pharmacol. 2025 Jun 12;16:1579613. doi: 10.3389/fphar.2025.1579613 (PMC12198167; doi:10.3389/fphar.2025.1579613)
Supplement: Supplementary file 1 [file DataSheet1.zip › Supplementary Material S9a.pdf]

Studies	Treatment	Toxicity			
		Grade 1*(n)*	Grade 2*(n)*	Grade 3*(n)*	Grade 4*(n)*
2003 Lin Jing	JFK Oral Liquid+Cisplatin (75mg/m2)	2 cases of Nausea/vomiting, 2 cases of constipation	1 case of constipation	None	None
	Cisplatin (75mg/m2)	2 cases of Leukopenia, 12 cases of hemoglobin decline, 7 cases of hemoglobin decline,7 cases of Nausea/vomiting, 7 cases of hair loss, 3 cases of constipation	7 cases of Leukopenia, 3 cases of hemoglobin decline, 4 cases of hemoglobin decline,8 cases of Nausea/vomiting, 8 cases of hair loss	11 cases of Leukopenia, 2 cases of hemoglobin decline, 1 cases of hemoglobin decline, 5 cases of Nausea/vomiting, 5 cases of hair loss	7 cases of Leukopenia, 3 cases of Nausea/vomiting
2011 Ding Ning	HS Oral Liquid + Cisplatin(80mg/m2)/Paclitaxel(80mg/m2)	23 cases of Leukopenia, 2 cases of thrombocytopenia, 13 cases of Nausea/vomiting	None	3 cases of Leukopenia, 2 cases of Nausea/vomiting	None
	Cisplatin(80mg/m2)/Paclitaxel(80mg/m2)	8 cases of thrombocytopenia, 2 cases of thrombocytopenia, 11 cases of Nausea/vomiting	None	2 cases of thrombocytopenia, 5 cases of Nausea/vomiting	None
2016 Pu Xiaolin	TFS(60mg/d) + Pembrolizumab(500mg/m2)	4 cases of Nausea/vomiting	None	None	None
	Pembrolizumab(500mg/m2)	5 cases of Nausea/vomiting	None	None	None
2017 Bai Haoran	JFK Oral Liquid+Cisplatin (75mg/m2) with Paclitaxel	6 cases of Leukopenia, 2 cases of thrombocytopenia, 3 cases of Nausea/vomiting, 2 cases of hair loss	8 cases of Leukopenia,2 cases of thrombocytopenia, 1 cases of Nausea/vomiting	None	None
	Cisplatin (75mg/m2) with Paclitaxel (175mg/m2)	7 cases of Leukopenia, 2 cases of Nausea/vomiting, 5 cases of constipation, 5 cases of anorexia	3 cases of Leukopenia, 1 case of Nausea/vomiting, 5 cases of constipation, 5 cases of anorexia	3 cases of Leukopenia	None
2019 Zhao Lei	TFS Oral Liquid+Cisplatin (75mg/m2)/Paclitaxel (175mg/m2)	None	None	None	None
	Cisplatin (75mg/m2) with Paclitaxel (175mg/m2)	1 cases of Fatigue, 2 cases of Nausea/vomiting, 1 cases of diarrhea, 2 cases of Proteinuria,1 cases of Leukopenia	None	1 case of Nausea/vomiting	None
2018 Tang MinLi	FZ Oral Liquid + Cisplatin (75mg/m2)	8 cases of fever, 1 case of Nausea/vomiting	None	None	None
	Cisplatin (75mg/m2)	10 cases of fever, 6 case of Nausea/vomiting	None	None	None
2018 Yang Li	HS Oral Liquid + Cisplatin(75mg/m2)/Paclitaxel(80mg/m2)	1 case of Nausea/vomiting, 6 cases of hair loss	None	None	None
	Cisplatin(75mg/m2)/Paclitaxel(80mg/m2)	4 cases of Nausea/vomiting, 8 cases of hair loss	2 cases of myelosuppression	None	None
2018 Zhang Junhong	JFK Oral Liquid + Cisplatin (75mg/m2)	6 cases of Nausea/vomiting,	None	None	None
	Cisplatin (75mg/m2)	8 cases of Nausea/vomiting, 8 cases of fever	None	None	None
2019 Zhai jianxia	HS Oral Liquid + Cisplatin (75mg/m2)	2 cases of Nausea/vomiting, 1 case of constipation	None	None	None
	Cisplatin (75mg/m2)	4 cases of Nausea/vomiting, 3 cases of constipation	None	None	None
2021 Xiao Lin	JFK Oral Liquid + Cisplatin (75mg/m2)	3 cases of Nausea/vomiting	None	None	None
	Cisplatin (75mg/m2)	2 cases of Nausea/vomiting	None	None	None
2021 Zhang Ren	JFK Oral Liquid + Cisplatin (75mg/m2)	9 cases of Leukopenia	6 cases of Leukopenia	None	None
	Cisplatin (75mg/m2)	20 cases of Leukopenia	9 cases of Leukopenia	None	None
