# Supplementary material for: Efficacy and safety of oral Chinese medicine combined with chemotherapy: a systematic review and network meta-analysis
Source: Front Pharmacol. 2025 Jun 12;16:1579613. doi: 10.3389/fphar.2025.1579613 (PMC12198167; doi:10.3389/fphar.2025.1579613)
Supplement: Supplementary file 1 [file DataSheet1.zip › Supplementary Material S9b.pdf]

Studies	Treatment	Toxicity			
		Grade 1*(n)*	Grade 2*(n)*	Grade 3*(n)*	Grade 4*(n)*
2011 Liu Yin	HS Oral Liquid + Cisplatin (80mg/m2) /Paclitaxel(80mg/m2)	1 case of Nausea/vomiting, 15 cases of hair loss	None	None	None
	Cisplatin (80mg/m2) /Paclitaxel(80mg/m2)	4 cases of Nausea/vomiting, 3 cases of myelosuppression, 26 cases of hair loss	None	None	None
2011 Xu Xinhua	HS Oral Liquid + Cisplatin (25mg/m2) /Paclitaxel(75mg/m2)	7 cases of Nausea/vomiting,12 cases of leukopenia,12 cases of thrombocytopenia	17 cases of Nausea/vomiting,17 cases of leukopenia,13 cases of thrombocytopenia	8 cases of Nausea/vomiting,6 cases of leukopenia,6 cases of thrombocytopenia	2 cases of Nausea/vomiting
	Cisplatin (25mg/m2) /Paclitaxel(75mg/m2)	5 case of Nausea/vomiting.7 cases of leukopenia,10 cases of thrombocytopenia	12 case of Nausea/vomiting,14 cases of leukopenia,15 cases of thrombocytopenia	14 case of Nausea/vomiting,11 cases of leukopenia,3 cases of thrombocytopenia	3 case of Nausea/vomiting,2 cases of leukopenia
2013 Zhang Wei	HS Oral Liquid + Cisplatin (80mg/m2) /Paclitaxel(80mg/m2)	1 case of Nausea/vomiting, 9 cases of hair loss	2 cases of Leukopenia	None	None
	Cisplatin (80mg/m2) /Paclitaxel(80mg/m2)	3 cases of Nausea/vomiting, 9 cases of hair loss	11 cases of Leukopenia	None	None
2017 Yang Wanlu	FZ Oral Liquid + Cisplatin (80mg/m2) /Paclitaxel(80mg/m2)	7 cases of myelosuppression, 10 cases of Nausea/vomiting	1 case of myelosuppression, 2 cases of Nausea/vomiting	None	None
	Cisplatin (80mg/m2) /Paclitaxel(80mg/m2)	9 cases of myelosuppression, 8 cases of Nausea/vomiting	5 case of myelosuppression, 7 cases of Nausea/vomiting	1 case of myelosuppression	None
2017 Yuan Xinxin	HS Oral Liquid + Cisplatin(75mg/m2)/Paclitaxel(80mg/m2)	16 cases of Nausea/vomiting, 12 cases of Leukopenia	5 cases of Nausea/vomiting, 5 cases of Leukopenia	1 case of Nausea/vomiting, 2 cases of Leukopenia	None
	Cisplatin(75mg/m2)/Paclitaxel(80mg/m2)	18 cases of Nausea/vomiting, 15 cases of Leukopenia	5 cases of Nausea/vomiting,7 cases of Leukopenia	1 case of Leukopenia	1 case of Leukopenia
2022 Song Li	TGT Oral Liquid + Cisplatin (75mg/m2) /Paclitaxel(175mg/m2)	17 cases of Nausea/vomiting, 10 cases of hair loss	None	None	None
	Cisplatin (75mg/m2) /Paclitaxel(175mg/m2)	20 cases of Nausea/vomiting, 12 cases of hair loss	None	None	None
2024 Zhao Weiwei	HS Oral Liquid + Cisplatin (50mg/m2) /Paclitaxel(260mg/m2)	8 cases of Nausea/vomiting, 4 cases of hair loss, 3 cases of myelosuppression	3 cases of Nausea/vomiting, 1 case of hair loss, 2 cases of myelosuppression	None	None
	Cisplatin (50mg/m2) /Paclitaxel(260mg/m2)	11 cases of Nausea/vomiting, 10 cases of hair loss, 4 cases of myelosuppression	14 cases of Nausea/vomiting, 15 cases of hair loss, 4 cases of myelosuppression	None	None
