# Supplementary figures and images for: Efficacy and safety of oral Chinese medicine combined with chemotherapy: a systematic review and network meta-analysis
Source: Front Pharmacol. 2025 Jun 12;16:1579613. doi: 10.3389/fphar.2025.1579613 (PMC12198167; doi:10.3389/fphar.2025.1579613)

a. OOR:


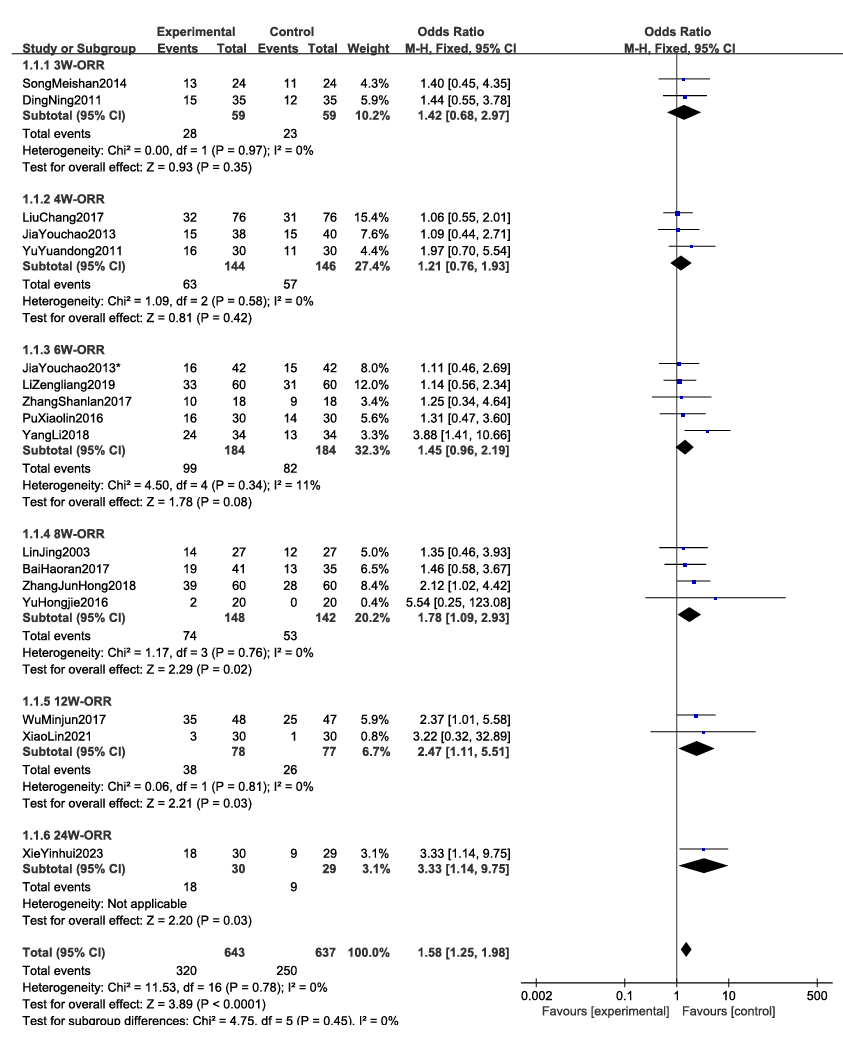


b. TP-ORR:


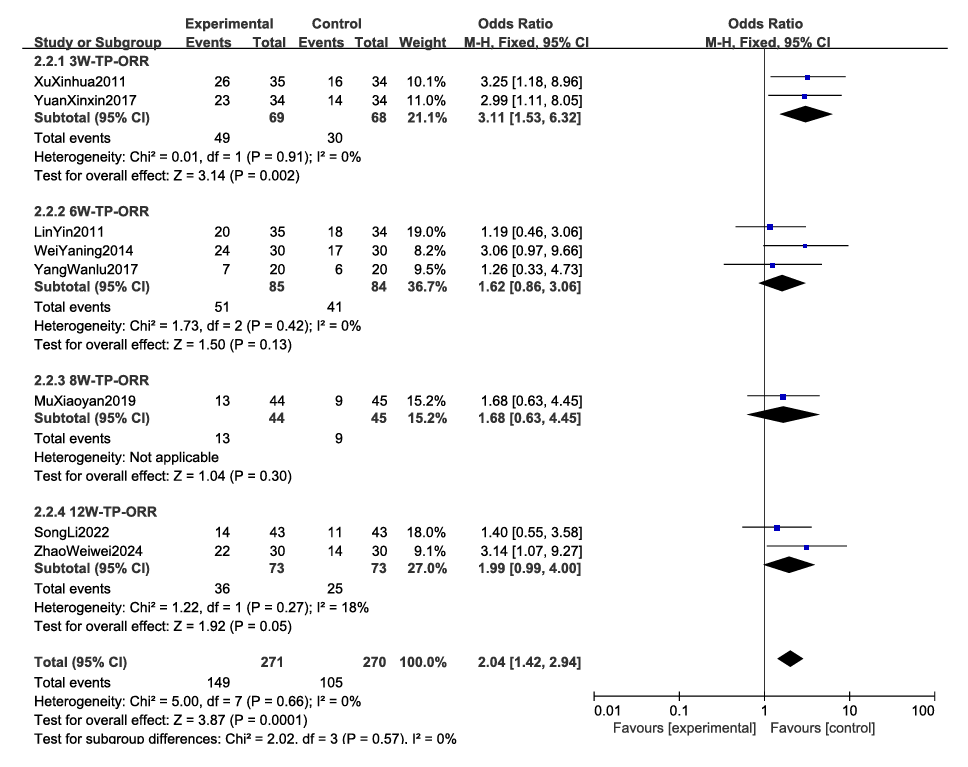

Supplement: Supplementary file 1 [file DataSheet1.zip › Supplementary Material S6.docx]

**TSA**

**ORR:**

**
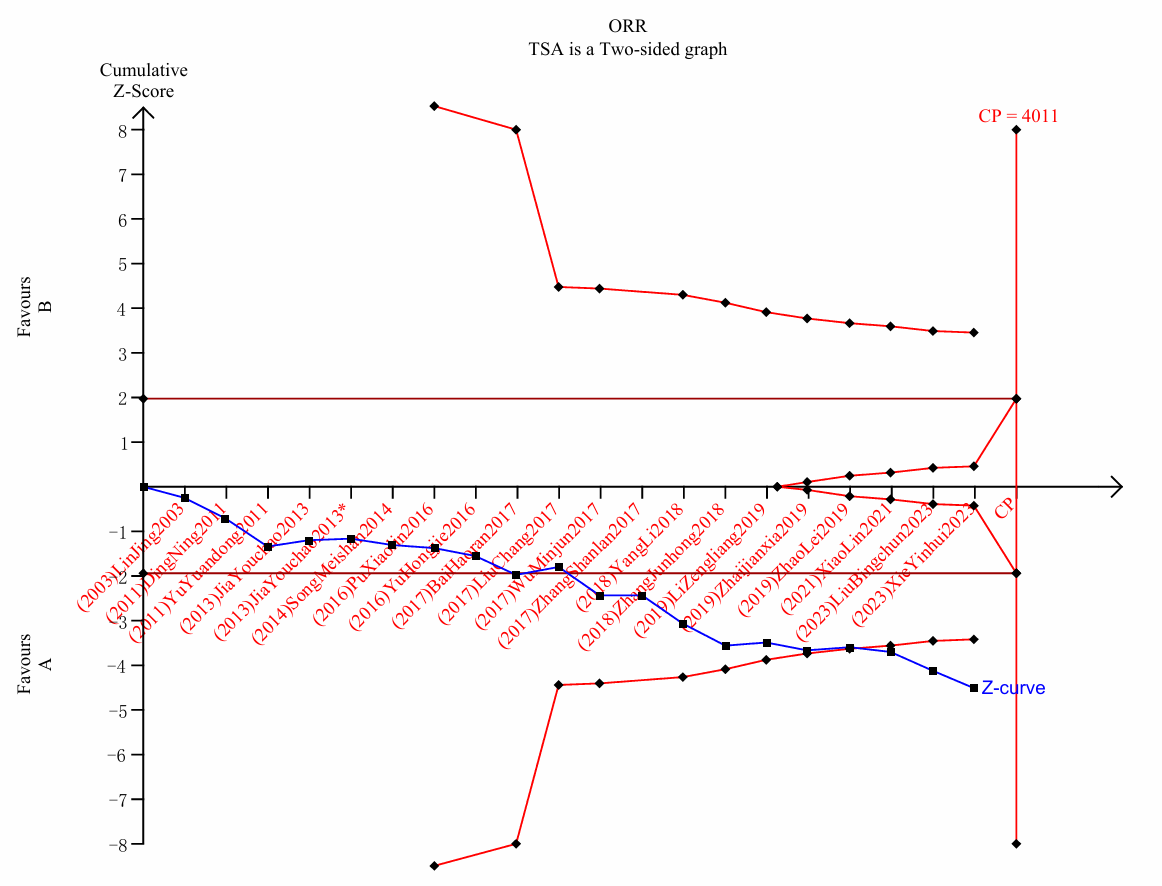
**

**TP-ORR:**

**
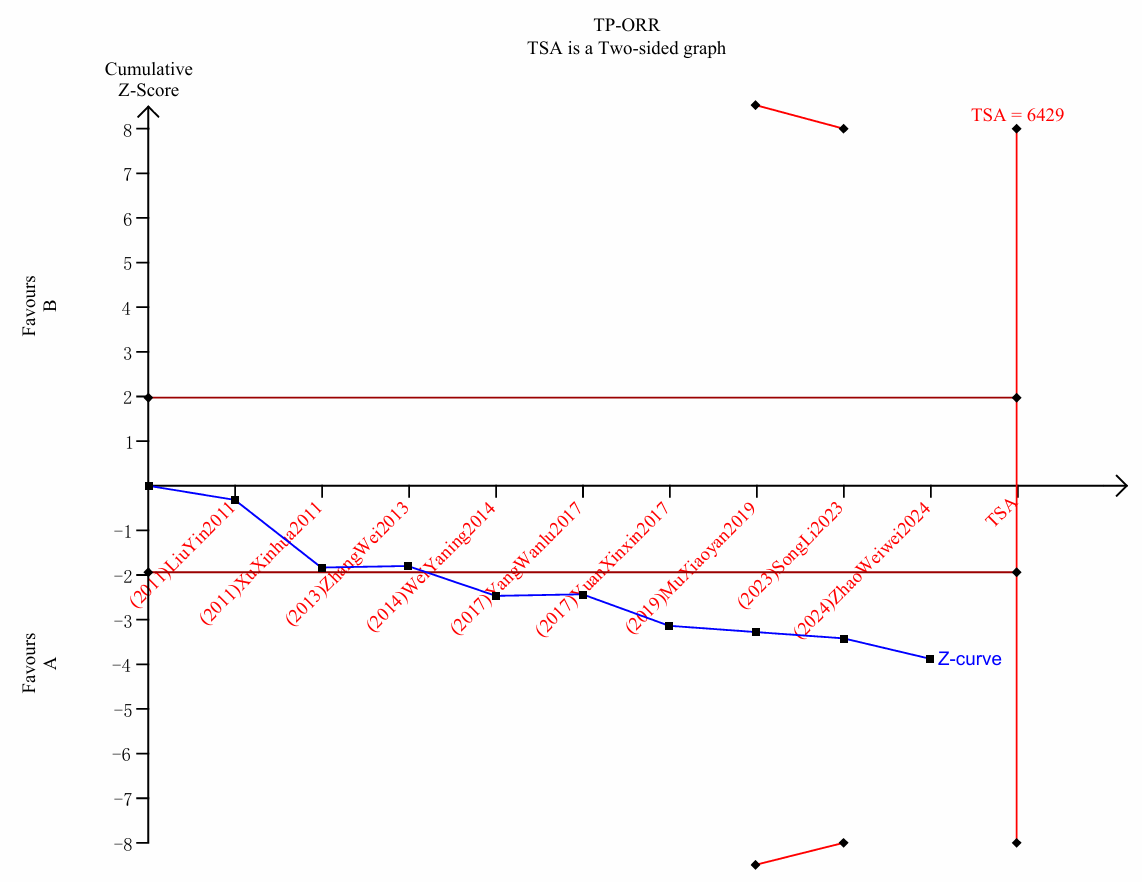
**

Supplement: Supplementary file 1 [file DataSheet1.zip › Supplementary Material S7.docx]

**Consistency test**

**OOR:
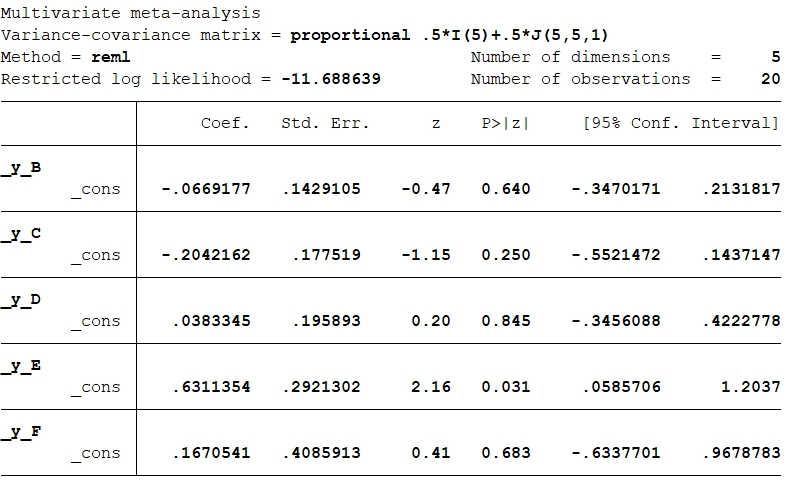
TP therapy:**

**
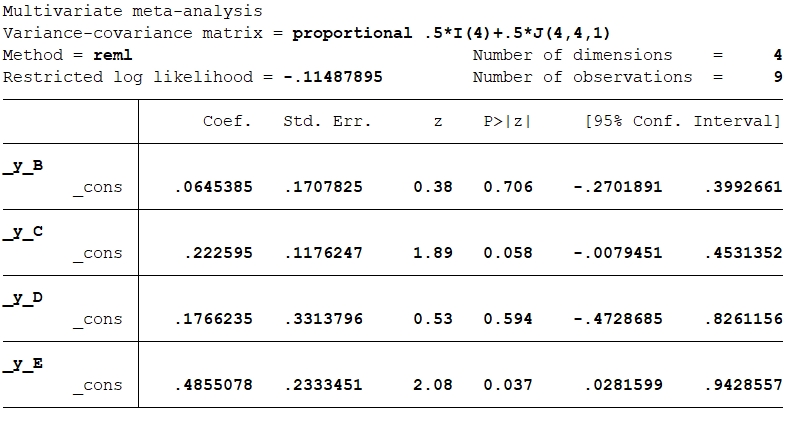
**

**TCM syndrome:**

**
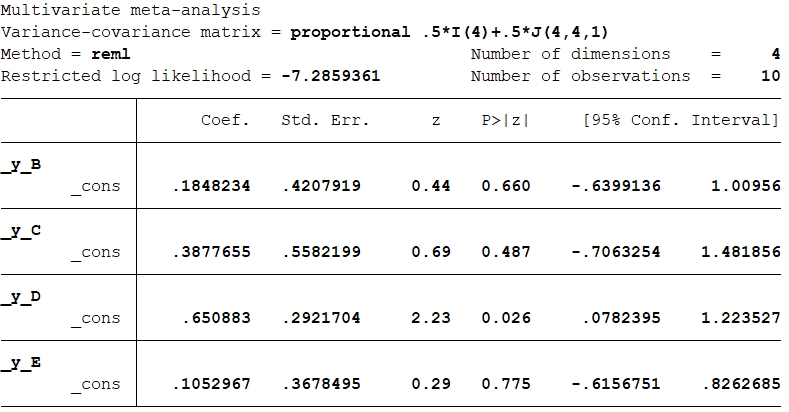
**

**CD4-CD8:**

**
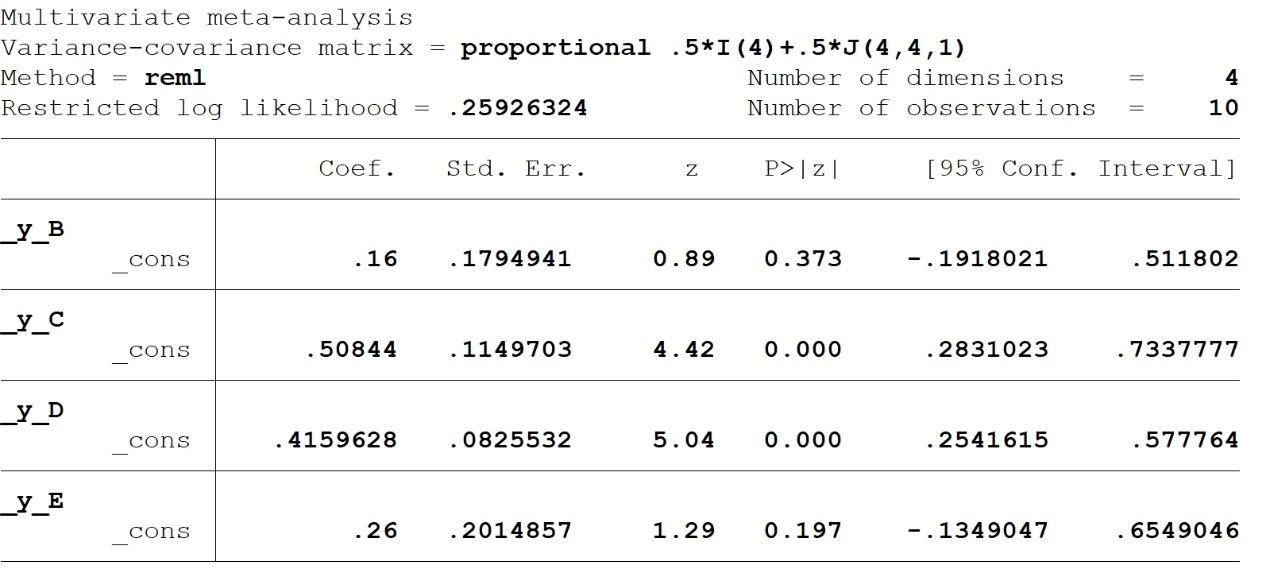
**

**NK:**

**
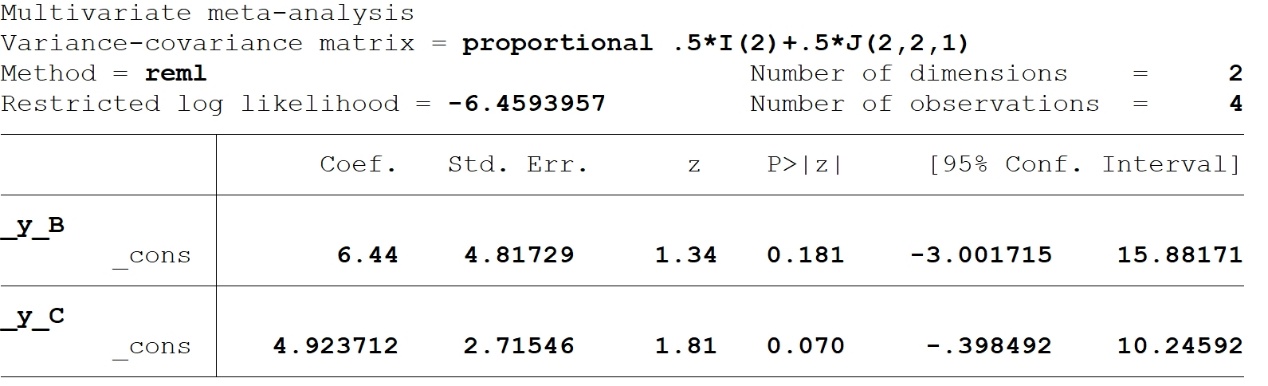
**

**CA125:**

**
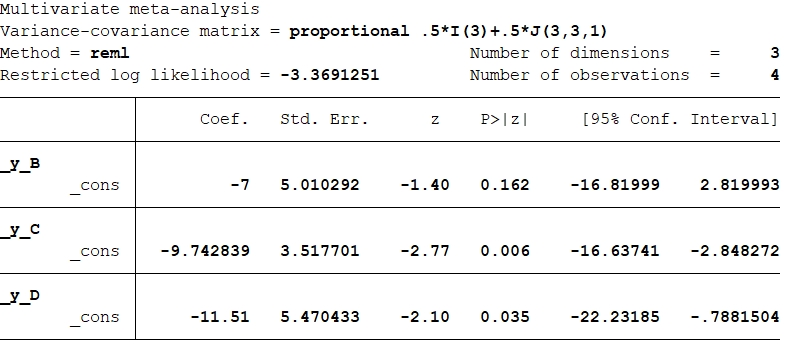
**

**CEA:**

**
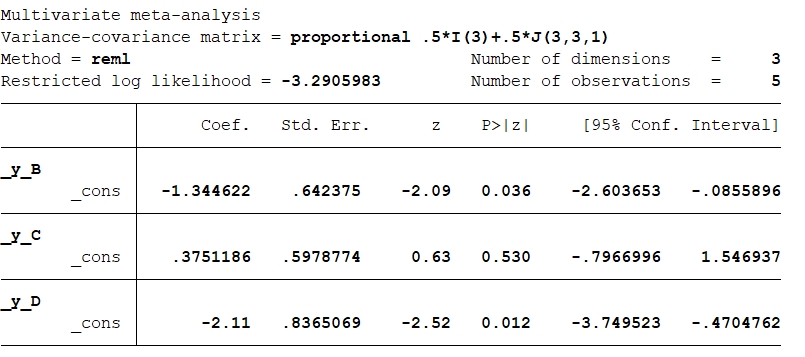
**

**CYFRA 21-1:**

**
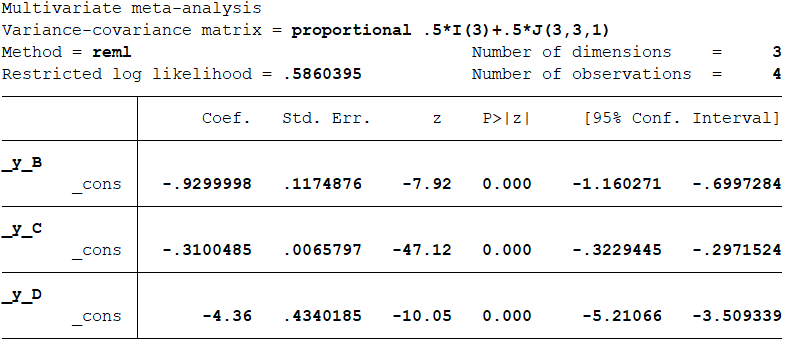
**

**Platelet:**

**
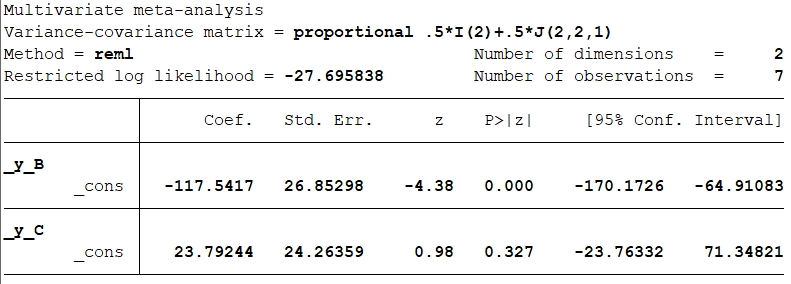
**

Supplement: Supplementary file 1 [file DataSheet1.zip › Supplementary Material S8.docx]
